# Highly asymmetric rice genomes

**DOI:** 10.1186/1471-2164-8-154

**Published:** 2007-06-08

**Authors:** Jing Ding, Hitoshi Araki, Qiang Wang, Pengfei Zhang, Sihai Yang, Jian-Qun Chen, Dacheng Tian

**Affiliations:** 1 State Key Laboratory of Pharmaceutical Biotechnology, Department of Biology, Nanjing University, Nanjing 210093, China; 2 Department of Zoology, Oregon State University, Corvallis, Oregon 97331, USA

## Abstract

**Background:**

Individuals in the same species are assumed to share the same genomic set. However, it is not unusual to find an orthologous gene only in small subset of the species, and recent genomic studies suggest that structural rearrangements are very frequent between genomes in the same species. Two recently sequenced rice genomes *Oryza sativa *L. var. Nipponbare and *O. sativa *L. var. 93-11 provide an opportunity to systematically investigate the extent of the gene repertoire polymorphism, even though the genomic data of 93-11 derived from whole-short-gun sequencing is not yet as complete as that of Nipponbare.

**Results:**

We compared gene contents and the genomic locations between two rice genomes. Our conservative estimates suggest that at least 10% of the genes in the genomes were either under presence/absence polymorphism (5.2%) or asymmetrically located between genomes (4.7%). The proportion of these "asymmetric genes" varied largely among gene groups, in which disease resistance (*R*) genes and the *RLK *kinase gene group had 11.6 and 7.8 times higher proportion of asymmetric genes than housekeeping genes (*Myb *and *MADS*). The significant difference in the proportion of asymmetric genes among gene groups suggests that natural selection is responsible for maintaining genomic asymmetry. On the other hand, the nucleotide diversity in 17 *R *genes under presence/absence polymorphism was generally low (average nucleotide diversity = 0.0051).

**Conclusion:**

The genomic symmetry was disrupted by 10% of asymmetric genes, which could cause genetic variation through more unequal crossing over, because these genes had no allelic counterparts to pair and then they were free to pair with homologues at non-allelic loci, during meiosis in heterozygotes. It might be a consequence of diversifying selection that increased the structural divergence among genomes, and of purifying selection that decreased nucleotide divergence in each *R *gene locus.

## Background

**Figure 1:**
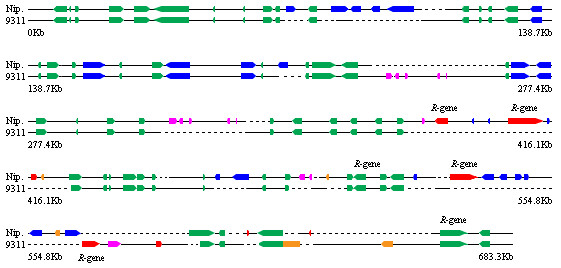
**An example of sequence comparison between two rice lines, Nipponbare and 93-11**. The region of 6,070,001–6,629,564 bp on chromosome 11 (based on IRGSP database) is shown. These 683 kb consecutive sequences, sampled around *R *gene clusters, were aligned between Nipponbare (559,563 bp) and 93-11 (428,114 bp). Dotted line represents where a sequence in one line cannot be aligned with a sequence at the corresponding position in the other line. Genes are shown as square boxes pointing in the direction of transcription. This region contains 70 non-TE genes in Nipponbare and 62 in 93-11. 53 allelic non-TE genes are colored as green, 8 P/A as red, 4 P/A_d _as orange, 14 AL as magenta, and 30 TE genes as blue boxes. All *R *genes are labeled above or below the boxes. The sizes of genes, intergenic sequences, and indels are proportionally scaled down. In this region, the PAG is 30.7%, and the percentage of asymmetric genes is 39.4% ((8 P/A +4 P/A_d _+14 AL)/66).

One usually expects that a gene found in an individual can be found in the others in the same species [1]. This expectation is based on an assumption of "stable" or "symmetric" genome structure within species. In human, for example, nucleotide sequences are highly identical among haplotypes [2]. However, recent genomic comparisons revealed that chromosomal rearrangements are quite common even within species [3]. Such rearrangements include insertion/deletion (indel), duplication and transposition. Some of the rearrangements contain more than 100 kb DNA [4]. Large rearrangements can produce either paralogous gene copies or indel polymorphisms of gene copy numbers. We define such genes as "asymmetric genes", which are located in insertion DNA, either present only in one genome (presence/absence, or P/A genes. Red genes in Figure 1) or located on different regions between genomes compared (asymmetrically located, or AL genes. Magenta genes in Figure 1). The mobilization of gene fragments from one location of the genome to another by Helitrons and the tandem gene amplifications following the whole-genome-duplication event are revealed to be general mechanisms for the insertion of noncollinear gene sequences in maize [5,6].

While mechanisms of these rearrangements have been extensively studied (e.g., [7,8]), little is known about the biological significance of asymmetric genes on genome evolution. Comparative studies on human genomes reveal a high level of variations in gene copy number and the resulting loss of gene products often cause genetic disease [4,9], indicating an evolutionary importance of asymmetric genes. In plants, indels of disease resistance (*R*) genes result in resistant and susceptible phenotypes to the infection of pathogens [10,11], and are subject to natural selection [12,13]. In addition, there are lines of evidence that indels play an important role in phenotypes of individuals in several ways, including an altered gene expression [14,15] and a noncollinearity in heterosis [1]. Gene duplication is another source of genetic variation [3,4]. A gene duplicated through a Helitron in some inbred lines of maize has been found to have an intact coding sequence, which is likely expressed conditionally [16]. These studies suggest that the asymmetrically existed or located genes may be an important molecular basis of many inheritable phenomena.

Yu et al. [17] found a relatively low proportion of P/A genes (2.2–3.3%) and a high proportion of intergenic nucleotide sequences that could not be aligned between two rice genomes, whereas Morgante et al. [18] found that 20% of genes are not shared between two inbred lines in maize. These results indicated that the proportion and the composition of asymmetric genes varied among species. A part of the reason, however, might be a different level of criteria used for detecting the asymmetric genes.

In this study, we comprehensively addressed the genomic asymmetry between two rice genomes, *Oryza sativa *L. var. Nipponbare and *O. sativa *L. var. 93-11, including both P/A and AL genes. The comparison of whole-genome sequences provided new insights into this issue. To investigate the proportion of asymmetric genes among intraspecific genomes, we systematically compared gene contents and locations between the two rice genomes. Furthermore, the proportions of asymmetric genes in four gene groups (*R *genes, the *RLK *kinase gene group, and two house-keeping gene groups, *Myb *and *MADS*) were examined to understand the evolution of asymmetric genes.

## Results

### Asymmetric genes between two rice genomes

Our selection of 2400 *indica *contigs contained 39.6 Mb non-overlapping sequences accounting for 10% of the whole rice genome (see Methods). Excluding doubtful contigs with possible misassembly or transposable elements (TE), 2094 contigs containing 34.4 Mb of 93-11 sequences were aligned with 34.7 Mb of Nipponbare sequences in total (Table 1). We counted all the indels of > 1 kb in the two genomes, and found 2.29 Mb inserts (6.6%) in Nipponbare and 1.78 Mb (5.2%) in 93-11. To reflect the proportion of DNA in the chromosomes that could not pair in meiosis in the heterozygote, the proportion of asymmetric genome (PAG) was defined as the total indel size divided by an average length of total DNA in the two genomes. The total PAG in 4.1 Mb of the two genomes was 11.8%. Based on TIGR or BGIs annotation [19,20], 132 and 197 functional genes were found in addition to 206 and 38 TE-related genes in Nipponbare and 93-11, respectively (Table 1; details in Table S1). A large portion (70.8% = 233/329, Table 1, 2) of the non-TE genes had motifs with known function (data not shown).

**Table 1:** Statistics of indels between Nipponbare and 93-11 genomes in 35 Mb sequences.

Cultivar	Inserts			Asymmetric genes in inserts			
		
	Length	No.	Total length (bp)	Known	Unknown	TE^a^	Total
Nipponbare	1–3 kb	213	306,912	31	4	10	45
(34.7 Mb	3–5 kb	79	316,675	17	3	26	46
aligned)	5–10 kb	97	629,958	15	17	65	97
	10–20 kb	58	752,324	23	12	80	115
	> 20 kb	9	287,930	7	3	25	35
Sub-total		456	2,293,799	93	39	206	338
93-11	1–3 kb	232	410,336	23	13	11	47
(34.4 Mb	3–5 kb	73	283,721	26	12	9	47
aligned)	5–10 kb	84	581,674	35	17	15	67
	10–20 kb	36	464,090	56	11	1	68
	> 20 kb	2	42,205	0	4	2	6
Sub-total		427	1,782,026	140	57	38	235
Total		883	4,075,825	**233**	96	244	573

Note. The bolded numbers are the same as the numbers in the bottom last column.

^a ^TE is transposable element related gene.

**Table 2:** Statistics of genes in genome, chromosome 10 and four gene groups.

Gene group^a^	Symmetric or asymmetric	Known function^b^	Multigene family^c^	Clustered genes^c^	Total
Inserts in Table 1					
In Nipponbare	P/A	13	3	1	20
	P/A_d_	43	56	8	56
	AL	37	46	11	56
In 93-11	P/A	45	1	0	62
	P/A_d_	23	35	6	35
	AL	72	36	9	100
	Total asy.-genes	233	177	35	329
Chromosome 10					
Genes in Nip.	Symmetric^d^			284	2,572
	P/A	40(18)	2(1)	0(0)	56(26)
	P/A_d_	49(31)	64(45)	2(1)	64(45)
	AL	32(23)	17(12)	2(2)	49(40)
	ND^e^				80
Genes in 93-11	Symmetric^d^				2,556
	P/A	46(30)	15(11)	5(4)	68(48)
	P/A_d_	49(40)	72(60)	2(2)	72(60)
	AL	37(24)	11(4)	1(0)	46(31)
	ND^e^				6
	Total asy.-genes	253(166)	181(133)	12(9)	355(250)
Gene family *R*-gene	Symmetric	339	35	132	339
	P/A	105	0	57	105
	P/A_d_	42	42	37	42
	AL	116	38	88	116
	Total asy.-genes	263	80	182	263
	Total				
*RLK*	Symmetric	264	99	111	264
	P/A	17	4	10	17
	AL	102	60	72	102
	Total asy.-genes	119	64	82	119
*Myb&MADS*	Symmetric	200	26	22	200
	P/A	6	0	ND	6
	AL	4	2	0	4
	Total asy.-genes	10	2	0	10

^a ^Asymmetric genes include P/A, P/A_d _and AL genes. The genes in unknown function, in single gene families and in non-clustered genes are equal to the total number of genes subtracts the genes in known function, in multigene families and in clustered genes, respectively.

^b ^The genes in the group of function known are determined by GO (gene ontology) classification.

^c ^The definition of multigene or clustered gene is described in Methods.

^d ^The symmetric genes between Nipponbare and 93-11 should be equal. However when an allele is a pseudogene, we still classify this gene into symmetric group because it has an allelic sequence. Therefore the numbers are not equal; the numbers in parentheses represent the P/A, P/A_d _and AL genes identified by the first method (see Methods for details), respectively. Otherwise the numbers stand for the asymmetric genes identified by the second method.

^e ^ND, not determined by the incomplete genome sequences (mainly in 93-11).

To distinguish the P/A genes from AL genes, all the asymmetric genes identified were subjected to the Blast search in the two genomes. Here we defined the P/A gene as an asymmetric gene for which no gene homologue of more than 20% gene coverage was found in another genome by the Blast search with the e value threshold < 0.10. If we found that a gene had an unequal number of homologues between genomes (e.g., 2 in Nipponbare and 3 in 93-11), the extra gene was categorized as a P/A duplicate (P/A_d_). The rest of asymmetric genes were defined as AL genes. Among the 329 non-TE genes, we identified 82 P/A, 91 P/A_d _and 156 AL genes (Table 2). The regularly sampled contigs allowed us to estimate a total of 3703 asymmetric genes between genomes (based on 389 Mb in the whole *japonica *genome; [21]), including 923 P/A, 1024 P/A_d _and 1756 AL genes. In total of 37544 non-TE genes in the *japonica *genome [21], the estimated percentages of P/A, P/A_d_, and AL genes are predicted to be 2.5%, 2.7%, and 4.7% between genomes, respectively. Namely, about 10% of the rice genes were asymmetric genes in the genomes. Note that the percentage of P/A genes was about a half of the estimate by Yu et al. (5.5% = 2.2% + 3.3%; [17]), suggesting that our estimation was very conservative.

### Potential biases involved in the estimation of P/A-gene number

To verify the proportion of asymmetric genes and their function using the newly-updated quality sequence and annotation [17,22], entire chromosome 10 assemblies of *japonica *(22.7 Mb, in August 2005) and *indica *genomes were chosen to perform the same analysis by two different criteria (see Methods for details). By the same criterion as used above, we found 9.1% of asymmetric genes in the total of 2741 genes in chromosome 10 (2.7% P/A, 3.8% P/A_d_, and 2.6% AL genes on different chromosomes, not including possible P/A_d _and AL genes on the same chromosome), confirming that our original estimates were reasonable (Table 2 and Table S1). Note that an asymmetric gene was identified only when no single piece of homologous sequence was found for > 20% gene coverage at the genome-wide Blast search with e value < 0.10 by this criterion.

In the second criterion, on the other hand, a homologous sequence was accepted when nucleotide similarity of entire homologous genes from two genomes was > 60% (CDS size ranges 150–7653 bp, 1048 bp on average). This quantitative criterion allowed us to precisely define an asymmetric gene. By this criterion, we could not find homologous sequences for some genes for which homologous sequences were found by the first criterion, and hence identified a higher percentage of asymmetric genes (13.0%), including 4.5% P/A, 5.0% P/A_d, _and 3.5% AL genes (not including possible P/A_d _and AL genes on the same chromosome, Table 2 and Table S1).

To evaluate potential bias in the result obtained by the second criterion, several factors were addressed. First, the sequence similarity was examined for all of the 50 P/A genes that had 20–45% homologous sequences in the other genome by the Blast search. This analysis revealed that these genes had no homologues with > 60% similarity for the entire gene sequences in the other genome, indicating that they are truly different genes and are present/absent between genomes.

Second, potential biases of TE-related genes were examined. Although we only used non-TE genes that were determined by Yuan et al. [22] in this study, all 355 asymmetric and 246 randomly-selected symmetric genes on chromosome 10 were examined in a local repeat database following Bennetzen et al. ([23]; see Methods for details) in order to confirm that they are truly non-TE genes. Both TE and non-TE genes might contain small parts of similar motifs or sequences. Therefore, the total proportion of TE-related fragments found in a gene was used to evaluate how many TE homologous sequences were contained in these genes. We found no significant difference of TE component between the asymmetric and symmetric genes (Table S2; χ^2^_Nipponbare _= 15.6 and χ^2^_93-11 _= 15.8, both *p *> 0.10). This analysis indicated no bias in containing TE homologous sequences between asymmetric and symmetric genes.

Third, we systematically evaluated the precision of the estimation of asymmetric-gene number. Our alignments of chromosome 10 in 93-11 by BLASTZ [24] covered 67.7% of Nipponbare sequences, which was close to the coverage 67.0% by International Rice Genome Sequencing Project (hereafter IRGSP; [21]). All genes in the alignable regions were symmetric except for a few AL genes (probably because of inversion or translocation on the same chromosome). In the remaining 32.3% of chromosome 10 in Nipponbare, 9.8% was identified as indel sequences (9.8% in Nipponbare and 15.3% in 93-11). Another 18.9% were non-alignable regions (Table S3; see Methods for definitions), which were presumably indels, too. The other 3.6% included 0.5% indels which were < 1 kb in both Nipponbare and 93-11, 0.2% filling DNA and 2.9% with unsequenced gaps in 93-11.

**Table 3:** Distribution of genes among rice populations or between species.

Rice lines		*R *Gene present in 9311 only											In Nipponbare only						In both lines-single gene								In both-multigene					Non-*R*		
				
Country	lines	1	2	3	4	5	6	7	8	9	10	11	12	13	14	15	16	17	18	19	20	21	22	23	24	25	26	27	28	29	30	31	32	33
Japan	Nipponbare	-	-	-	-	-	-	-	-	-	-	-	+	+	+	+	+	+	+	+	+	+	+	+	+	+	+	+	+	+	+	+	+	+
China	9311	+	+	+	+	+	+	+	+	+	+	+	-	-	-	-	-	-	+	+	+	+	+	+	+	+	+	+	+	+	+	+	+	+
USA	Calrose	-	-	-	-	-	+	-	-	-	-	-	+	+	+	+	+	+	+	+	+	+	+	+	+	+	+	+	+	+	+	+	+	+
USA	S4542	-	-	+	+	-	+	-	-	-	-	-	-	-	-	+	+	-	-	+	+	+	+	+	+	+	+	+	+	+	+	+	+	+
Haiti	Grassy	+	-	-	-	-	+	-	-	-	-	-	-	-	-	+	+	-	-	+	-	+	+	+	+	+	+	+	+	+	+	+	+	+
Guyana	Vintula	+	-	-	+	-	+	-	-	-	-	-	+	+	-	+	-	-	+	+	+	+	+	+	+	+	+	+	+	+	+	+	+	+
Zaire	CI	+	+	-	+	-	+	+	+	-	-	+	-	+	-	+	+	+	+	+	+	+	+	+	+	+	+	+	+	+	+	+	+	+
Tanzania	WC	-	-	-	+	-	-	-	-	-	-	-	-	+	+	+	+	+	+	+	+	+	+	+	+	+	+	+	+	+	+	+	+	+
Japan	Beiguang	+	-	-	+	-	-	-	-	-	-	-	-	+	+	+	+	+	+	+	+	+	+	+	+	+	+	+	+	+	+	+	+	+
South Korea	Milyang46	+	+	+	+	+	+	+	+	+	-	+	-	-	-	-	-	-	+	+	+	+	+	+	+	+	+	+	+	+	+	+	+	+
India	Dular	+	-	-	-	-	-	+	+	+	-	-	-	+	-	+	-	-	-	+	+	+	+	+	+	+	+	+	+	+	+	+	+	+
Philippines	IR24	+	+	+	+	+	+	-	-	+	+	+	+	-	-	+	-	-	+	+	+	+	+	+	+	+	+	+	+	+	+	+	+	+
China	NJ16	+	-	+	+	-	+	+	+	+	+	+	+	+	-	-	-	-	+	+	+	+	+	+	+	+	+	+	+	+	+	+	+	+
China	Dadaotou	-	-	-	+	-	-	-	-	-	-	-	+	+	-	+	+	+	+	+	+	+	+	+	+	+	+	+	+	+	+	+	+	+
China	Zhenshan97	+	+	+	-	+	+	+	-	+	-	-	-	+	-	-	-	-	+	-	-	+	+	+	+	+	+	+	+	+	+	+	+	+
China	Yunjing	-	-	+	-	-	+	-	-	-	-	-	-	+	-	+	+	-	+	+	+	+	+	+	+	+	+	+	+	+	+	+	+	+
China	C416	-	+	-	-	+	+	-	-	+	-	-	-	-	-	+	+	-	+	+	+	+	+	+	+	+	+	+	+	+	+	+	+	+
China	PA64	-	-	+	+	-	+	+	+	+	-	-	-	-	-	+	-	-	+	-	-	+	+	+	+	+	+	+	+	+	+	+	+	+
Wild Rice	S03005	+	-	+	+	+	+	+	+	+	+	+	+	+	-	+	+	+	+	+	+	+	+	+	+	+	+	+	+	+	+	+	+	+
Wild Rice	S01169	-	+	-	+	+	+	-	-	-	-	+	-	+	-	+	+	+	+	+	+	+	+	+	+	+	+	+	+	+	+	+	+	+
Present frequency%		55	35	45	65	30	75	40	35	45	20	40	35	65	20	80	60	40	85	90	85	100	100	100	100	100	100	100	100	100	100	100	100	100
π value (‰)		2.4	2.0	12	1.3	0.7	48	2.8	0.6	0.0	0.0	12	0.0	0.5	0.0	2.9	0.0	0.0	38	13	0.8	2.0	1.8	2.1	4.0	0.0	135	2.9	79	74	41	0.0	5.0	2.8^a^
Related sp.	*0. offcinalis*	-	-	-	-	+	-	-	-	-	-	-	-	+	-	-	+	+	-	+	-	-	+	+	+	+	+	-	-	-	+	+	+	+
Related sp.	*0. meyeriana*	-	-	-	-	-	-	+	-	-	-	-	-	+	-	-	+	+	-	+	+	-	-	+	-	-	-	-	+	-	-	+	+	+

Note. + stands for the presence of gene, and – for absence of gene suggested by PCR and sequencing; Gene 1-30 are *R *genes, and 31-33 are house keeping genes. The accession number or name of gene 1-33 is OsIFCC009208, OsIFCC035754, OsIFSC047991, OsIFCC019895, OsIFCC035751, OsIFCC035229, OsIFCC043050, OsIFCC045542, OsIFCC023444, OsIFCC026523, OsIFSC046156, Os12g31620, Os11g45330, Os06g16790, Os12g09730, Os10g04670, Os11g11550, Os12g28250, Os11g15670, Os01g20720, Os12g18360 (*Pi-ta*), Os04g41370, Os07g29820, Os02g25900, OsIFCC040819, Os12g37270 and Os12g37280 (*Pib *homologue), Os12g03750 (OsIFCC032042 and OsIFCC032029 in 93-11), Os01g57270 (and Os01g57280, Os01g57310, Os01g57340), Os05g16180 (and Os05g16200, Os11g47780), Os08g10430 (and Os08g10440), Os01g05490 (triosephosphate isomerase), Os03g61120 (anthranilate synthase), and Os05g49760 (isocitrate dehydrogenase), respectively.

^a^*π*, valued as 1/1000, denotes the average nucleotide diversity and the values for the five multigene families and three house keeping genes were calculated from allelic pairs between Nipponbare and 93-11.

Fourth, we compared the gene percentages with empirically-validated genes, the expressed or functional genes (with EST or conserved motifs), between symmetric and asymmetric genes. A similar percentage (about 70%) was observed, which suggested that the 'real' genes showed the same pattern in both symmetric and asymmetric genes.

Finally, to examine a possibility of overestimating the P/A-gene number due to un-sequenced gaps of genomes, we checked all 82 P/A genes that have small pieces of homologous sequences in the Blast search. If a homologous piece was not included in a gene or a contig of another genome, this piece was likely to be located in a gap. If a piece was included in a gene or contig, on the other hand, this gene was likely true P/A genes, based on the fact that 97.7% of genes could be found in at least one piece in either 93-11 or Nipponbare [17]. Only six P/A genes were found to be in the first category (not likely a true P/A). All these results suggested that the real proportion of asymmetric genes in rice genomes was probably > 10%, and particularly that the asymmetric DNA was likely > 20%, whereas there were both under- and over-estimating factors for the proportion of asymmetric genes.

In addition, the sequence comparison between GLA4 (an *indica *variety with BAC-based sequences available in chromosome 4, [25]) and Nipponbare (2.2 Mb) also provided an estimate of a 20.6% (449.9 kb) of indels (> 1 kb) and 43 non-TE genes in indels (or 12.5% asymmetric genes), which again showed a similar rate of asymmetric DNA (or genes) between rice genomes.

### Variation of asymmetric genes among gene groups

A gene that has essential functions to the organism will be conserved in a genome by natural selection. For genes that have functions to cope with the variable environmental factors (e.g., the genes to recognize the highly variable molecules of pathogens), on the other hand, different gene compositions may be preferable among genomes [26,27]. If this is the case, the amount of asymmetric genes may vary among gene groups with different functions.

To find out the distributions of asymmetric genes among different gene groups, four gene groups were selected for a genome-wide analysis between Nipponbare and 93-11. One was the *R *genes representing gene groups under diversifying selection [26,28] and possibly with high proportions of asymmetric genes [26]. Another was the *RLK *genes containing a functional cytoplasmic kinase domain and playing roles in diverse processes in growth and development [29]. The other two were house-keeping gene groups, *Myb *and *MADS*, which were involved in plant development and cell cycle regulation [30,31]. In *R *genes, 483 genes from 93-11 and 461 genes from Nipponbare were identified including 105 P/A (62 in 93-11 and 43 in Nipponbare), 42 P/A_d _(21 in 93-11 and 21 in Nipponbare), and 116 AL (58 in each genome) genes (Table S1). These were 22.2%, 8.9%, and 24.6% of *R *genes, respectively. 319 *RLK *genes included 17 (5.3%) P/A and 102 (32.0%) AL genes. On the other hand, 208 *Myb *and *MADS *included only 6 (2.9%) P/A and 4 (1.9%) AL genes. In total, 55.7% of *R *genes, 37.3% of *RLK*, and 4.8% of housekeeping genes were asymmetric. Comparing with the genome-wide average of the asymmetric structure (9.9%), these percentages were 5.6 times higher in *R *genes and 3.8 times higher in *RLK *genes, whereas housekeeping genes showed a lower percentage of asymmetry (48% of the genome-wide average). *R *genes and *RLK *genes had 11.6 and 7.8 times higher proportions of asymmetric genes than housekeeping genes in rice genomes. These differences were highly significant (χ^2 ^= 119, *p *< 10^-5^), suggesting that the proportion of asymmetric genes is not at random and affected by natural selection.

The extremely high proportion of asymmetric *R *genes could also be demonstrated by the 0.6 Mb consecutive sequences (Figure 1), which contained two *R *gene clusters. Sequence comparison at this region on chromosome 11 (560 kb in Nipponbare and 428 kb in 93-11) demonstrated the genomic arrangement of asymmetric genes, which revealed 30.7% of PAG and 39.4% of asymmetric genes in this region.

### Origin of asymmetric genes

Indels can be generated by unequal crossing-over in clustered genes [32] or by insertion of transposable elements [5,33]. The genes generated by different factors will show different makeup of asymmetric genes. For example, Morgante et al. [18] showed that many of the asymmetric makeup were caused by the activity of nonautonomous helitrons, whereas Jiang et al. [34] described that the gene fragments were ferried around the rice genome by transposition. Therefore, the proportion of clustered (including tandemly-arranged) or TE-related genes among asymmetric genes could provide clues for the origin of asymmetric DNA. To address this issue, we grouped asymmetric genes into clustered and non-clustered genes or single and multigene families (Table 2; see Methods for definitions). The percentage of clustered asymmetric genes in total asymmetric genes was only 2.4% (4/169) in Nipponbare chromosome 10. This percentage was much smaller than 11.0% (284/2572), the number of clustered allelic genes divided by that of total allelic genes in the same chromosome (Table 2). Similarly, this percentage was not high in 93-11 chromosome 10 (4.3% = 8/186) and in genome-wide samples (10.6% = 35/329, Table 2). Among gene groups, however, the percentages varied largely. The percentage of clustered genes was 69.2% in asymmetric genes and 38.9% in allelic genes in *R *genes, whereas these percentages were 63.9% and 41.8% in *RLK*, and 0% and 11.0% in housekeeping genes. The same trend was observed in the comparisons between the percentage of multigenes in asymmetric genes and that of allelic genes (Table 2). These were 30.4% and 10.3% in *R *genes, 53.8% and 37.5% in *RLK*, and 20.0% and 13.0% in housekeeping genes. These results indicated the clustered genes (or genes in multigene families), which were presumably created by duplication, did not particularly contribute to the composition of asymmetric genes in entire genomes but might contribute to the composition of some specific gene groups.

Obviously, TE-related genes composed a major part (60.9% = 206/338) of asymmetric genes in Nipponbare (Table 1). This percentage was twice as much of 27.4%, the portion of TE-related genes (14196; [19]) amongst the total genes in this genome, suggesting that the asymmetric DNA (inserted DNA) in Nipponbare may be a part of genome expansion caused by repeat-elements. This percentage was very different in 93-11 (16.2%, Table 1). The low percentage in 93-11 indicated that the evolutionary importance of TE elements in asymmetric DNA might vary among genomes. However, this percentage might be biased because of the shot-gun sequencing strategy and the selected long contigs in 93-11, which would lead to the enrichment of less repetitive regions in 93-11.

### Variation of P/A-R genes among populations

**Figure 2:**
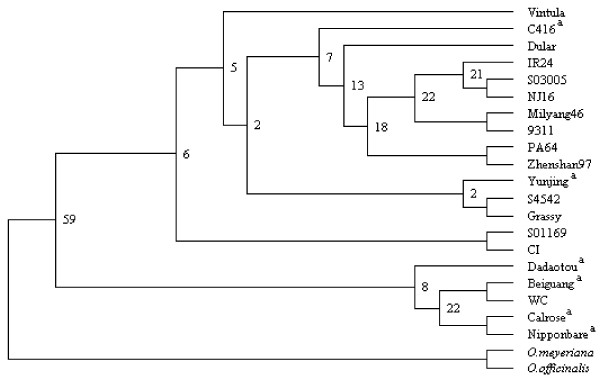
**Grouping of *R *genes in Table 3 among rice strains and closely related species**. This tree was created based on the discrete morphology (parsimony) method using the programs SEQBOOT, PARS and CONSENSE of the PHYLIP package v3.6. Percent bootstrap values from 1000 replicate samples are indicated at all the major nodes. ^a ^indicates *japonica *rice.

We used the *R *gene group to verify the existence of P/A genes among rice populations. The genotyping of 18 *O. sativa *lines and 2 *O. rufipogon *ecotypes from worldwide populations confirmed that all of the 17 P/A genes were under the present/absent polymorphism. The presence-allele frequencies varied largely (20–80%, Table 3), as also shown in *Arabidopsis *[13]. Phylogenetic analysis showed no sign of geographic differentiation and *japonica-indica *sub-specific differentiation (Figure 2). These results suggested that the high proportion of asymmetric *R *genes observed between 93-11 and Nipponbare was commonly asymmetric in rice populations. Interestingly, 3 of the 8 single genes (37.5%) found both in Nipponbare and 93-11 (Table 3) were not fixed among the other lines, suggesting that even more *R *genes can be P/A genes among populations.

The average nucleotide diversity among alleles in rice lines for 17 P/A-*R *genes was 0.0051 (0–0.048, Table 3). Majority of P/A-*R *genes showed very low genetic diversity. In two related species (*O. officinalis *and *O. meyeriana*, Table 3), most of the P/A genes (12/17) could not be found (Table 3). The other 5 genes, which could be found in these species as inter-specific orthologs, showed extremely low nucleotide diversities among rice lines (0.0008 on average, Table 3).

Although a failure of detecting PCR products did not necessarily mean an absence of homologous gene, there were three reasons to believe that *R *gene homologues were really missing in some lines. First, two positive controls (multi-*R *genes and non-*R *genes), one with higher nucleotide diversity and another with a similar level of diversity, always succeeded in detecting PCR products (Table 3). Second, most of the selected *R *genes represented low genetic diversities (0.0051 on average), indicating that these genes were not highly variable. Finally, we tried multiple PCR methods with lower stringencies for amplification (see Methods for details) when PCR products were not detected in some strains. Thus, at least most of the cases, these *R *genes must be under the presence/absence polymorphism in this species.

## Discussion

### Estimation and significance of asymmetric genes in plant

Our manual alignments of sequences allowed us to estimate 5.2% P/A (including P/A_d_) and 4.7% AL genes between two rice genomes. These must be underestimations because of our conservative methods. Our analysis on the chromosome 10 provided a good example, in which 9.5% P/A genes (4.5% of P/A and 5.0% of P/A_d_) were identified, and AL genes could be more than 10%. The 3.5% AL genes identified in different chromosomes did not include the AL genes on the same chromosome (5.2%; 67 in Nipponbare and 75 in 93-11, data not shown) and the undetermined genes (3.1%; 86 these genes in Table 2, in which no counterpart could be identified in current database). Though some of these AL genes could not be precisely determined by current sequence data, there was a good reason to assume that the majority of them were asymmetric genes. Therefore, the total percentage of AL genes could be > 10%.

The significant proportion of asymmetric genes identified in this study was well consistent with the results from previous studies. For example, the percentage of P/A genes (4.5%) was close to that of P/A genes identified by Yu et al. (5.5%; [17]), and our results for the proportion of asymmetric genes (13.0%) and asymmetric DNA (> 20%) was comparable to the higher proportion (25%) of asymmetric DNA indicated between these two genomes [17]. The 2.1 and 2.2 Mb map-based BAC cloning sequences between rice cultivar GLA4 and Nipponbare revealed a 20.6% of indels (> 1 kb) and 12.5% asymmetric genes in indels. The dramatic structural differences between maize inbred lines were also found in the *bz *[1] and *z1C *genomic region [15]. Although the DNA-sequence noncollinearity in these regions has been attributed to retrotransposon insertions due to the gain of pseudogenes from another donor site in the genome [35,36], the genome-wide survey suggested that there are up to 10,000 P/A genes in maize genomes [18]. Furthermore, large variations in genome size were evident within many species. For example, DNA content varied up to 10% among *Arabidopsis *populations [37], 12% between soybean lines [38], and 50% between maize lines [39]. In the alignable rice genome sequences, the total size of DNA segments slightly varied at 1.0% (34.66 vs. 34.46 Mb) or 5.6% (2.245 vs. 2.125 Mb) between Nipponbare and 93-11 or GLA4. However the PAG ranged from 11.8% to 20.6% between rice genomes. These results indicated that the high PAG within species was common in plants.

It was obvious that single nucleotide polymorphisms (SNPs) were important in genetic variation [40]. However, the proportion of SNP in a genome was much smaller than PAG. For example in the homologous sequences of Nipponbare and GLA4, PAG was 53 times higher than the proportion of SNP (0.39%; [25]) in the same region. Although it did not necessarily reflect a relative biological importance of SNPs to PAG, PAG could contribute to unpaired homologous chromosome segments in meiosis in heterozygotes, and hence to genomic reorganizations that had large impacts on genomic evolution [41]. This was a similar effect to 'hybrid-dysfunction' at the level of chromosomal rearrangement [42]. The estimated number of asymmetric genes in total (3703) was remarkably large among rice genomes, and the number among maize genomes was probably even larger [18,39]. Our analyses on the gene groups suggested that the asymmetric genes were affected by natural selection, and were probably playing important roles in many inheritable phenomena, such as heterosis, inbreed depression, and disease resistance [1,10,11].

The noncollinearity of *z1C *gene cluster among maize inbred lines provides a good example. The same genomic interval containing this gene cluster among lines significantly lost their gene collinearity and also differed in the regulation of each remaining gene set [5,14,15]. The alteration of expression patterns led to an effect of "overdominance" which could contribute to a heterosis phenomenon. Given the fact that chromosomes could consist of intervals of haplotypes that are highly diverged, endless breeding opportunities could be predicted, because of their linear arrangement along a chromosome and their expression potential in hybrid combinations [5,14,15]. The altered gene expression in hybrids by such noncollinearity could promote neofunctionalization.

### Maintenance of asymmetric genes

The high proportion of asymmetric genes in genomes could be simply driven by the random genetic drift. Thus, the presence/absence polymorphism itself could be only a transit stage. If this was the case, the origin of the polymorphism must be relatively young [43]. In this scenario, evolutionary rates of asymmetric genes must be similar among different gene groups. On the other hand, if the polymorphism was maintained by diversifying selection, as suggested in *R *genes in *Arabidopsis *[12,13], the origin of the polymorphism must be old and the evolutionary rate could be different among gene groups. The age of the polymorphism in P/A genes could be estimated by examining the level of nucleotide diversity among rice lines relative to the level of genetic divergence between species [44]. The nucleotide diversities in the 17 P/A-*R *genes was generally low (*π *= 0–0.048, average 0.0051). The low genetic diversities for majority of the observed genes indicated that these genes were either young or under purifying selection. If the P/A-*R *genes were duplicated relatively recently, similar paralogs should be found in the genomes. However, no similar paralogs for any P/A-*R *genes were found based on a conserved NBS region in *R*-genes either in Nipponbare or in 93-11 (data not shown), indicating that young duplications cannot explain the abundance of P/A-*R *genes. The old P/A-*R *genes with low nucleotide diversity might be a consequence of diversifying selection that preferred a large numbers of diverse P/A-*R *genes for plant-pathogen interaction, and of purifying selection that suppressed nucleotide diversities by functional constraint on each P/A-*R *genes. *Rpm1*, the first discovered P/A-*R *gene in *Arabidopsis *[10], was a good example. This gene was genetically almost identical between populations, but still appeared to be under presence/absence polymorphism in distantly-related species [44]. Due to its highly conserved sequence and ancient origin, *Rpm1 *was believed to be under purifying selection on gene itself and under balancing selection to maintain a stable frequency among populations [12,44].

### Evolutionary dynamics of asymmetric genes

Ceaseless insertion, deletion, translocation and unequal recombination will contribute to the increase of asymmetric DNA. Any of these genomic rearrangements will create higher-level of intraspecific violations of genetic collinearity [5]. The asymmetric DNA may have a self-enhancing mechanism by generating instability in a heterozygote because the free DNA has higher chances to pair selectively with homologues at non-allelic loci (or paralogs). A higher rate of unequal crossing-over could be the consequence. In a heterozygote such as the hybrid between Nipponbare and 93-11, there are 55.7% of *R *genes that has no orthologous alleles to pair with during meiosis, and the high rate of unpaired DNA would increase the asymmetric DNA through the high-frequency unequal crossing-over. The other factors, such as inbreeding, fixation and gene loss, and purifying selection, may also contribute to the genomic symmetry. On the other hand, the genomic asymmetry must be balanced with genetic stability. Although it is no doubt that vast majority of symmetric alleles are the basis of stable inheritance, the asymmetric architecture of genomes should also be a core of genetic variation and genomic evolution. The ratio of symmetric to asymmetric DNA in genomes may reflect the balance between stable inheritance and variation required for the species.

## Methods

### Alignment of genomic sequences

In the database of 93-11 genome [20], the large pieces of sequences available as of October 2004 were the contig 1–10000, ranging from 8–60 kb in length [45]. To obtain the contigs with full ranges in sizes of > 8 kb, we chose 2400 contigs with the number of 1–240 in last three digits. Each of the selected contigs was subjected to BLASTN search (e value threshold < 0.10) against Nipponbare genome. The contigs with no hit in the Blast search were excluded to avoid any possible misassembly, as we could not determine their locations. The rest of the contigs was aligned with their Nipponbare counterparts manually by Sequencher 4.1 (Gene Codes Corp., Ann Arbor MI). When an indel (> 1 kb) was present in an alignment, Clustal X [46] was used to exclude any alignment error. When the end sequence of a contig could not align with its Nipponbare sequence, it was excluded to avoid any possible misassembly in 93-11. 2.1 Mb and 2.2 Mb map-based BAC cloning sequences in GenBank for rice cultivar GLA4 [25] and Nipponbare, respectively, were also used to exclude any other misassembly.

### Gene annotation and data analysis

Genes in insertion sequences were identified based on the annotation of TIGR or BGIs. All non-TE genes were examined in the local repeat database, based on Oryza Repeat Database [47], using BLASTN searches as described by Bennetzen et al. [23] to detect any TE sequences in the coding region of genes. After removing the TE-related genes, the association of genes were performed using GOst [48] to find out their ontologies. The CDS of each predicted gene in inserts was searched for homologues in the local database of whole Nipponbare and 93-11 genomic sequences using BLASTN (e value threshold < 0.10).

### Identification of asymmetric genes on chromosome 10

Two methods were used to identify the asymmetric genes on chromosome 10. In the first method, all non-TE genes on chromosome 10 (annotated by TIGR; [22]) and complete sequences in this chromosome of Nipponbare [19] and 93-11 [20] were included into our local database on August 2005. Each CDS of these genes was used for cross-blast searches (e value threshold < 0.10) in the local sequence database of Nipponbare and 93-11. If a gene in a genome had sequence counterparts (> 20% in length in total) for its CDS in another genome, this gene was determined to have an allele in another genome. If no counterparts (or < 20% in length in total) were found in another genome or in chromosome 10 of another genome, this gene was identified as an asymmetric gene. Then all asymmetric genes were subjected to the Blast search against all genomic sequences (including syngenta database) to find out whether the gene was a P/A, P/A_d _or AL gene by its copies in either one or both of these genomes.

In the second method, the complete sequences of chromosome 10 in Nipponbare (22.68 Mb covering 96.6% of euchromatic regions; [21]) and 93-11 (20.7 Mb) were aligned by BLASTZ [24]. This program is designed for identifying orthologous regions and for alignments of long genomic sequences. We used the same scoring matrix as the one used for pairwise alignments of human and chimpanzee [24]. To minimize false-positive alignments, only alignments with > 300 bp were maintained and any > 300 bp non-alignable region remained as gap. The search for asymmetric genes focused on the non-alignable regions. Two overlapping strategies were used for cross-search. First we tried to identify the true insertions (> 1 kb) in both genomes. If the sequences flanking an insertion of Nipponbare matched an unbroken sequence of 93-11 and the filling DNA (the non-alignable region) in 93-11 was < 1 kb, or vice versa, an insertion was defined (Table S3). The other non-alignable regions (> 1 kb in both genomes) could not be determined as insertions (named as non-alignable regions). Second, all the CDS of genes in insertions and non-alignable regions were subjected to the Blast search (e value threshold < 0.10) against the whole genome sequences of Nipponbare (both IRGSP and syngenta database) and 93-11. If no counterpart in blast search was found, or the total length of counterparts was < 50% of the CDS and the nucleotide similarity of entire counterpart gene was < 60%, it was assigned as a P/A gene. The P/A_d _or AL was determined by their copies in either or both of these genomes by the same criteria as used for P/A genes.

### Identification of four gene groups

NBS-LRR (nucleotide-binding site-leucine-rich repeat) genes, a common type of *R *genes [27], were chosen to represent the most polymorphic *R*-group [26]. The genes encoding a cytoplasmic serine/threonine (ser/thr) protein kinase, a single-pass transmembrane domain and an extracellular leucine-rich repeat domain were defined as *RLK *gene group [49]. *MADS *and *Myb *genes contained either *MADS*-box or *Myb *domain, both of which were DNA binding domains [30,31]. The same methods as Yang et al. [50] were employed to identify the *R *genes in genomes of rice cultivars, Nipponbare and 93-11. Each *R *gene with its flanking sequences was subjected to the Blast search against genomic sequences of both 93-11 and Nipponbare to find out all the possible homologues (< 30% nucleotide diversity) and to locate their physical positions in chromosomes. Homologous sequences were manually aligned by Sequencher v.4.1. The methods employed to identify genes in the groups of *RLK*, *Myb *and *MADS *were as described in Shiu and Bleecker [49], Davidson et al. [30], and Parenicova et al. [31].

### Identification of gene clusters and gene families

We defined a clustered gene if two or more homologous genes resided within 80 kb. A criterion, < 30% of nucleotide diversity, was used to define homologues within a family. Each asymmetric gene was subjected to the Blast search against the local database of Nipponbare and 93-11 to find all possible homologues. If more than one homologue were found in Nipponbare or 93-11, the homologues were designated as genes in a multi-gene family.

### Plant materials

Eighteen lines of world-wide *O. sativa*, two ecotypes of wild rice *O. rufipogon *(S03005 and S01169) and two related species (*O. officinalis *and *O. meyeriana*) were chosen to detect the presence/absence of *R *genes among these populations. The rice lines were obtained from USDA, National Plant Germplasm System, USA and Dr. Cailin Wang in Institute of Food Crops, Jiangsu Academy of Agricultural Sciences, China, and wild rice and related species from Dr. Dajian Pan in National Guangzhou Wild-Rice Conservation, China.

### Genotyping and sequencing

All the lines were genotyped by a PCR method. The primer pairs were designed to amplify ~700 bp fragments in the LRR region (Table S4). We investigated the presence/absence of 17 P/A genes (11 in 93-11 and 6 in Nipponbare) using 14 allelic (symmetric) genes (8 in single gene and 6 in multigene families) as positive controls, which were randomly chosen from the genes presented in both Nipponbare and 93-11. Three housekeeping genes were also selected as another positive control. To obtain reliable results, the PCR products were sequenced to verify the expected genes amplified (GenBank: EF533726-EF533871). In cases of null PCR products, we repeated the PCR reactions two more times with one at 5°C lower annealing temperature (45–50°C); if no PCR products were obtained again, we repeated the PCR reaction once more using a newly designed primer pair for the genes. For the gene -3, -6, -11, -18 and -19 (Table 3), we used conserved regions based on the sequence information from various rice strains because their average nucleotide diversities (*π *in Table 3) were > 0.01. We believed that these efforts maximized excluding false PCR results due to heterogenic sequences.

## Authors' contributions

Jing Ding: AB, ES

Hitoshi Araki: ES

Qiang Wang: MT

Pengfei Zhang: MT

Sihai Yang: MT

Jian-Qun Chen: ES

Dacheng Tian: ES, FG

All authors read and approved the final manuscript.

## Supplementary Material

Additional file 1 Table S1 Click here for file

Additional file 2 Table S2–S4 Click here for file
