# Supplementary material for: Highly asymmetric rice genomes
Source: BMC Genomics. 2007 Jun 8;8:154. doi: 10.1186/1471-2164-8-154 (PMC1914357; doi:10.1186/1471-2164-8-154)
Supplement: Additional file 1 — Table S1 [file 1471-2164-8-154-S1.pdf]

Table S1: Asymmetric genes on chromosome 10, aligned contigs and four gene families.

Number of gene or contig	type of Asymmetric genes	Chromo -some	GO Categories or function	Single or multigene family	Single or clustered locus
**Genes on chromosome 10 of Nipponbare**					
LOC_Os10g03680	PA	10	C[a]	single	non-cluster
LOC_Os10g08050	PA	10	C&P	single	non-cluster
LOC_Os10g05560	PA	10	F&P	single	non-cluster
LOC_Os10g03580	PA	10	unknown	single	non-cluster
LOC_Os10g04650	PA	10	C&F&P	single	non-cluster
LOC_Os10g37000	PA	10	unknown	single	non-cluster
LOC_Os10g36300	PA	10	unknown	single	non-cluster
LOC_Os10g04630	PA	10	unknown	single	non-cluster
LOC_Os10g04670	PA	10	F&P	single	non-cluster
LOC_Os10g02720	PA	10	C&F&P	single	non-cluster
LOC_Os10g42930	PA	10	unknown	single	non-cluster
LOC_Os10g30320	PA	10	C	single	non-cluster
LOC_Os10g41840	PA	10	C	multigene	non-cluster
LOC_Os10g03500	PA	10	C	single	non-cluster
LOC_Os10g36330	PA	10	C	single	non-cluster
LOC_Os10g06150	PA	10	unknown	single	non-cluster
LOC_Os10g03100	PA	10	F	single	non-cluster
LOC_Os10g20360	PA	10	unknown	single	non-cluster
LOC_Os10g04660	PA	10	unknown	single	non-cluster
LOC_Os10g29440	PA	10	F	single	non-cluster
LOC_Os10g02890	PA	10	C&P	single	non-cluster
LOC_Os10g12190	PA	10	C	single	non-cluster
LOC_Os10g22710	PA	10	C	single	non-cluster
LOC_Os10g07050	PA	10	C	single	non-cluster
LOC_Os10g25370	PA	10	C&F&P	single	non-cluster
LOC_Os10g41990	PA	10	C&P	single	non-cluster
LOC_Os10g34010	PA	10	C&F&P	single	non-cluster
LOC_Os10g09200	PA	10	C	single	non-cluster
LOC_Os10g02270	PA	10	unknown	single	non-cluster
LOC_Os10g01340	PA	10	C&F&P	single	non-cluster
LOC_Os10g05770	PA	10	C	single	non-cluster
LOC_Os10g26450	PA	10	C&F&P	single	non-cluster
LOC_Os10g05890	PA	10	C	single	non-cluster
LOC_Os10g09560	PA	10	unknown	single	non-cluster
LOC_Os10g29350	PA	10	C	single	non-cluster
LOC_Os10g37090	PA	10	unknown	single	non-cluster

LOC_Os10g31260	PA	10	C&F&P	single	non-cluster
LOC_Os10g31300	PA	10	C	single	non-cluster
LOC_Os10g29430	PA	10	unknown	single	non-cluster
LOC_Os10g35500	PA	10	F	single	non-cluster
LOC_Os10g29410	PA	10	F	single	non-cluster
LOC_Os10g10140	PA	10	C	single	non-cluster
LOC_Os10g33070	PA	10	C	single	non-cluster
LOC_Os10g04210	PA	10	F	single	non-cluster
LOC_Os10g36970	PA	10	C&F&P	single	non-cluster
LOC_Os10g09360	PA	10	F	single	non-cluster
LOC_Os10g03510	PA	10	unknown	single	non-cluster
LOC_Os10g12040	PA	10	C	single	non-cluster
LOC_Os10g20870	PA	10	F&P	single	non-cluster
LOC_Os10g03590	PA	10	unknown	single	non-cluster
LOC_Os10g02330	PA	10	unknown	single	non-cluster
LOC_Os10g04680	PA	10	unknown	single	non-cluster
LOC_Os10g07280	PA	10	C	single	non-cluster
LOC_Os10g27000	PA	10	F&P	single	non-cluster
LOC_Os10g33100	PA	10	C	single	non-cluster
LOC_Os10g08170	PA	10	C	multigene	non-cluster
LOC_Os10g11100	AL	10	C	multigene	non-cluster
LOC_Os10g09150	AL	10	C	multigene	non-cluster
LOC_Os10g24930	AL	10	C	multigene	non-cluster
LOC_Os10g03410	AL	10	C	single	non-cluster
LOC_Os10g34230	AL	10	C	single	non-cluster
LOC_Os10g34260	AL	10	C	single	non-cluster
LOC_Os10g30690	AL	10	C	single	non-cluster
LOC_Os10g34250	AL	10	C&P	multigene	non-cluster
LOC_Os10g04360	AL	10	F	multigene	non-cluster
LOC_Os10g05830	AL	10	unknown	multigene	cluster
LOC_Os10g04380	AL	10	unknown	single	non-cluster
LOC_Os10g16930	AL	10	unknown	single	non-cluster
LOC_Os10g05900	AL	10	unknown	multigene	non-cluster
LOC_Os10g16890	AL	10	unknown	single	non-cluster
LOC_Os10g05610	AL	10	unknown	single	non-cluster
LOC_Os10g29250	AL	10	unknown	single	non-cluster
LOC_Os10g42040	AL	10	unknown	single	non-cluster
LOC_Os10g30700	AL	10	unknown	single	non-cluster
LOC_Os10g35900	AL	10	unknown	multigene	non-cluster
LOC_Os10g34240	AL	10	unknown	single	non-cluster
LOC_Os10g34280	AL	10	unknown	multigene	non-cluster
LOC_Os10g26820	AL	10	unknown	single	non-cluster
LOC_Os10g20640	AL	10	unknown	multigene	non-cluster
LOC_Os10g26790	AL	10	unknown	single	non-cluster

LOC_Os10g40240	AL	10	unknown	multigene	non-cluster
LOC_Os10g26440	AL	10	unknown	single	non-cluster
LOC_Os10g34270	AL	10	C	single	non-cluster
LOC_Os10g12620	AL	10	C&F	single	non-cluster
LOC_Os10g09240	AL	10	C&F	single	non-cluster
LOC_Os10g01570	AL	10	C&F&P	single	non-cluster
LOC_Os10g05160	AL	10	C&F&P	single	non-cluster
LOC_Os10g03420	AL	10	C&F&P	multigene	non-cluster
LOC_Os10g05630	AL	10	C&F&P	single	non-cluster
LOC_Os10g05170	AL	10	C&F&P	single	non-cluster
LOC_Os10g01580	AL	10	C&F&P	single	non-cluster
LOC_Os10g04100	AL	10	C&F&P	multigene	non-cluster
LOC_Os10g34870	AL	10	C&F&P	multigene	non-cluster
LOC_Os10g18030	AL	10	C&F&P	single	non-cluster
LOC_Os10g34220	AL	10	C&F&P	multigene	non-cluster
LOC_Os10g34300	AL	10	C&F&P	single	non-cluster
LOC_Os10g36310	AL	10	C&F&P	single	non-cluster
LOC_Os10g05410	AL	10	C&P	multigene	cluster
LOC_Os10g05180	AL	10	C&P	single	non-cluster
LOC_Os10g24900	AL	10	F	single	non-cluster
LOC_Os10g34310	AL	10	F	single	non-cluster
LOC_Os10g26940	AL	10	F	single	non-cluster
LOC_Os10g05600	AL	10	F&P	multigene	non-cluster
LOC_Os10g13380	AL	10	F&P	single	non-cluster
LOC_Os10g04370	AL	10	P	single	non-cluster
LOC_Os10g02590	PA_d	10	unknown	multigene	non-cluster
LOC_Os10g07060	PA_d	10	C	multigene	non-cluster
LOC_Os10g10740	PA_d	10	C	multigene	non-cluster
LOC_Os10g11610	PA_d	10	C	multigene	non-cluster
LOC_Os10g40560	PA_d	10	C	multigene	non-cluster
LOC_Os10g03890	PA_d	10	C	multigene	non-cluster
LOC_Os10g29040	PA_d	10	C	multigene	non-cluster
LOC_Os10g03520	PA_d	10	C	multigene	non-cluster
LOC_Os10g40770	PA_d	10	C	multigene	non-cluster
LOC_Os10g25730	PA_d	10	C	multigene	non-cluster
LOC_Os10g08640	PA_d	10	C	multigene	non-cluster
LOC_Os10g04200	PA_d	10	C	multigene	non-cluster
LOC_Os10g04350	PA_d	10	C	multigene	non-cluster
LOC_Os10g30980	PA_d	10	C	multigene	non-cluster
LOC_Os10g07130	PA_d	10	C	multigene	non-cluster
LOC_Os10g30970	PA_d	10	C	multigene	non-cluster
LOC_Os10g02680	PA_d	10	C&F&P	multigene	non-cluster
LOC_Os10g24950	PA_d	10	C&P	multigene	non-cluster
LOC_Os10g11590	PA_d	10	C&P	multigene	non-cluster

LOC_Os10g10750	PA_d	10	C&P	multigene	non-cluster
LOC_Os10g02870	PA_d	10	C&P	multigene	non-cluster
LOC_Os10g18700	PA_d	10	C&P	multigene	non-cluster
LOC_Os10g03390	PA_d	10	F&P	multigene	cluster
LOC_Os10g11600	PA_d	10	unknown	multigene	non-cluster
LOC_Os10g04260	PA_d	10	unknown	multigene	non-cluster
LOC_Os10g40980	PA_d	10	unknown	multigene	non-cluster
LOC_Os10g04080	PA_d	10	unknown	multigene	non-cluster
LOC_Os10g06810	PA_d	10	unknown	multigene	non-cluster
LOC_Os10g29060	PA_d	10	unknown	multigene	non-cluster
LOC_Os10g04800	PA_d	10	unknown	multigene	non-cluster
LOC_Os10g01830	PA_d	10	unknown	multigene	non-cluster
LOC_Os10g01400	PA_d	10	unknown	multigene	non-cluster
LOC_Os10g05920	PA_d	10	unknown	multigene	non-cluster
LOC_Os10g19820	PA_d	10	unknown	multigene	non-cluster
LOC_Os10g30230	PA_d	10	unknown	multigene	non-cluster
LOC_Os10g18850	PA_d	10	unknown	multigene	non-cluster
LOC_Os10g09890	PA_d	10	unknown	multigene	non-cluster
LOC_Os10g12180	PA_d	10	unknown	multigene	non-cluster
LOC_Os10g42030	PA_d	10	C&F	multigene	non-cluster
LOC_Os10g06860	PA_d	10	C&F&P	multigene	non-cluster
LOC_Os10g03210	PA_d	10	C&F&P	multigene	non-cluster
LOC_Os10g04280	PA_d	10	C&F&P	multigene	non-cluster
LOC_Os10g07120	PA_d	10	C&F&P	multigene	non-cluster
LOC_Os10g12750	PA_d	10	C&F&P	multigene	non-cluster
LOC_Os10g10790	PA_d	10	C&F&P	multigene	non-cluster
LOC_Os10g12090	PA_d	10	C&F&P	multigene	non-cluster
LOC_Os10g21570	PA_d	10	C&F&P	multigene	non-cluster
LOC_Os10g37080	PA_d	10	C&F&P	multigene	non-cluster
LOC_Os10g21500	PA_d	10	C&F&P	multigene	non-cluster
LOC_Os10g26850	PA_d	10	C&F&P	multigene	non-cluster
LOC_Os10g24960	PA_d	10	C&F&P	multigene	non-cluster
LOC_Os10g20820	PA_d	10	C&P	multigene	non-cluster
LOC_Os10g26430	PA_d	10	F	multigene	non-cluster
LOC_Os10g08970	PA_d	10	F	multigene	non-cluster
LOC_Os10g34860	PA_d	10	F	multigene	non-cluster
LOC_Os10g30680	PA_d	10	F	multigene	non-cluster
LOC_Os10g12140	PA_d	10	F	multigene	non-cluster
LOC_Os10g03360	PA_d	10	F&P	multigene	cluster
LOC_Os10g11620	PA_d	10	F&P	multigene	non-cluster
LOC_Os10g10730	PA_d	10	F&P	multigene	non-cluster
LOC_Os10g03400	PA_d	10	F&P	multigene	non-cluster
LOC_Os10g29280	PA_d	10	F&P	multigene	non-cluster
LOC_Os10g11200	PA_d	10	F&P	multigene	non-cluster

LOC_Os10g12170	PA_d	10	P	multigene	non-cluster
**Genes on chromosome 10 of 93-11**					
OsIFCC031095	PA	10	unknown	single	non-cluster
OsIFCC031622	PA	10	unknown	multigene	cluster
OsIFCC029730	PA	10	unknown	single	non-cluster
OsIFCC029978	PA	10	unknown	single	non-cluster
OsIFCC030641	PA	10	unknown	multigene	cluster
OsIFCC031584	PA	10	unknown	single	non-cluster
OsIFCC030785	PA	10	unknown	multigene	non-cluster
OsIFCC030194	PA	10	C	single	non-cluster
OsIFCC029714	PA	10	C	single	non-cluster
OsIFCC030962	PA	10	C	single	non-cluster
OsIFCC031373	PA	10	C	single	non-cluster
OsIFCC044308	PA	10	C	single	non-cluster
OsIFCC029782	PA	10	C&F	multigene	non-cluster
OsIFCC029896	PA	10	C&P	single	non-cluster
OsIFCC030659	PA	10	C&P	single	non-cluster
OsIFCC029756	PA	10	C&P	single	non-cluster
OsIFCC044130	PA	10	F&P	multigene	non-cluster
OsIFCC029754	PA	10	F&P	single	non-cluster
OsIFCC030510	PA	10	F&P	single	non-cluster
OsIFCC029923	PA	10	unknown	multigene	non-cluster
OsIFCC030638	PA	10	unknown	multigene	cluster
OsIFCC030642	PA	10	unknown	multigene	cluster
OsIFCC029755	PA	10	unknown	multigene	non-cluster
OsIFCC030000	PA	10	unknown	single	non-cluster
OsIFCC030591	PA	10	unknown	single	non-cluster
OsIFCC030607	PA	10	unknown	multigene	non-cluster
OsIFCC030710	PA	10	unknown	single	non-cluster
OsIFCC030779	PA	10	unknown	single	non-cluster
OsIFCC030795	PA	10	unknown	single	non-cluster
OsIFCC031123	PA	10	unknown	single	non-cluster
OsIFCC031125	PA	10	unknown	single	non-cluster
OsIFCC031364	PA	10	unknown	single	non-cluster
OsIFCC044077	PA	10	unknown	single	non-cluster
OsIFCC044164	PA	10	unknown	single	non-cluster
OsIFCC044239	PA	10	unknown	single	non-cluster
OsIFCC031019	PA	10	C	single	non-cluster
OsIFCC044348	PA	10	C	single	non-cluster
OsIFCC029924	PA	10	C&F	multigene	non-cluster
OsIFCC031268	PA	10	C&F&P	single	non-cluster
OsIFCC030146	PA	10	C&F&P	multigene	non-cluster
OsIFCC044368	PA	10	C&F&P	single	non-cluster
OsIFCC029757	PA	10	C&F&P	single	non-cluster

OsIFCC029930	PA	10	C&F&P	single	non-cluster
OsIFCC030072	PA	10	C&F&P	single	non-cluster
OsIFCC030377	PA	10	C&F&P	single	non-cluster
OsIFCC030737	PA	10	C&F&P	single	non-cluster
OsIFCC030784	PA	10	C&F&P	single	non-cluster
OsIFCC030835	PA	10	C&F&P	single	non-cluster
OsIFCC031124	PA	10	C&F&P	single	non-cluster
OsIFCC044059	PA	10	C&F&P	single	non-cluster
OsIFCC044177	PA	10	C&F&P	single	non-cluster
OsIFCC044319	PA	10	C&F&P	single	non-cluster
OsIFCC030868	PA	10	C&P	single	non-cluster
OsIFCC044520	PA	10	C&P	single	non-cluster
OsIFCC031376	PA	10	C&P	multigene	cluster
OsIFCC030281	PA	10	F	single	non-cluster
OsIFCC031474	PA	10	F&P	single	non-cluster
OsIFCC029781	PA	10	F&P	single	non-cluster
OsIFCC029693	PA	10	F&P	single	non-cluster
OsIFCC029753	PA	10	F&P	single	non-cluster
OsIFCC030067	PA	10	F&P	single	non-cluster
OsIFCC030069	PA	10	F&P	single	non-cluster
OsIFCC030388	PA	10	F&P	single	non-cluster
OsIFCC030637	PA	10	F&P	single	non-cluster
OsIFCC031090	PA	10	F&P	single	non-cluster
OsIFCC031091	PA	10	F&P	single	non-cluster
OsIFCC031627	PA	10	F&P	multigene	non-cluster
OsIFCC029922	PA	10	F&P	multigene	non-cluster
OsIFCC031201	AL	10	unknown	multigene	non-cluster
OsIFCC031623	AL	10	unknown	single	non-cluster
OsIFCC029919	AL	10	C	single	non-cluster
OsIFCC031422	AL	10	C	multigene	cluster
OsIFCC044584	AL	10	C	multigene	non-cluster
OsIFCC029729	AL	10	C	single	non-cluster
OsIFCC029928	AL	10	C	multigene	non-cluster
OsIFCC030598	AL	10	C	single	non-cluster
OsIFCC030707	AL	10	C	single	non-cluster
OsIFCC044250	AL	10	C	multigene	non-cluster
OsIFCC044329	AL	10	C	single	non-cluster
OsIFCC031586	AL	10	C&F&P	single	non-cluster
OsIFCC030708	AL	10	C&P	multigene	non-cluster
OsIFCC044392	AL	10	C&P	multigene	non-cluster
OsIFCC030433	AL	10	F&P	single	non-cluster
OsIFCC031017	AL	10	unknown	single	non-cluster
OsIFCC029713	AL	10	unknown	single	non-cluster
OsIFCC030108	AL	10	unknown	single	non-cluster

OsIFCC030869	AL	10	unknown	single	non-cluster
OsIFCC031348	AL	10	unknown	single	non-cluster
OsIFCC031571	AL	10	unknown	single	non-cluster
OsIFCC044274	AL	10	unknown	single	non-cluster
OsIFCC044284	AL	10	unknown	single	non-cluster
OsIFCC044375	AL	10	C	multigene	non-cluster
OsIFCC044247	AL	10	C	single	non-cluster
OsIFCC044288	AL	10	C	single	non-cluster
OsIFCC044074	AL	10	C	multigene	non-cluster
OsIFCC044327	AL	10	C	single	non-cluster
OsIFCC030883	AL	10	C&F	single	non-cluster
OsIFCC030758	AL	10	C&F&P	multigene	non-cluster
OsIFCC029887	AL	10	C&F&P	single	non-cluster
OsIFCC029888	AL	10	C&F&P	single	non-cluster
OsIFCC029889	AL	10	C&F&P	single	non-cluster
OsIFCC029890	AL	10	C&F&P	single	non-cluster
OsIFCC029892	AL	10	C&F&P	single	non-cluster
OsIFCC029929	AL	10	C&F&P	single	non-cluster
OsIFCC030101	AL	10	C&F&P	single	non-cluster
OsIFCC044326	AL	10	C&F&P	single	non-cluster
OsIFCC044401	AL	10	C&F&P	single	non-cluster
OsIFCC044583	AL	10	C&F&P	single	non-cluster
OsIFCC030870	AL	10	C&P	single	non-cluster
OsIFCC029796	AL	10	F	multigene	non-cluster
OsIFCC029790	AL	10	F	single	non-cluster
OsIFCC030432	AL	10	F&P	single	non-cluster
OsIFCC031632	AL	10	F&P	single	non-cluster
OsIFCC030100	AL	10	P	single	non-cluster
OsIFCC029850	PA_d	10	F&P	multigene	non-cluster
OsIFCC029947	PA_d	10	unknown	multigene	non-cluster
OsIFCC030031	PA_d	10	F&P	multigene	non-cluster
OsIFCC030060	PA_d	10	C&P	multigene	non-cluster
OsIFCC030107	PA_d	10	C	multigene	non-cluster
OsIFCC030276	PA_d	10	F&P	multigene	non-cluster
OsIFCC030280	PA_d	10	C&F&P	multigene	non-cluster
OsIFCC030362	PA_d	10	unknown	multigene	non-cluster
OsIFCC030709	PA_d	10	unknown	multigene	non-cluster
OsIFCC030772	PA_d	10	unknown	multigene	non-cluster
OsIFCC030796	PA_d	10	C	multigene	non-cluster
OsIFCC031282	PA_d	10	C	multigene	non-cluster
OsIFCC031283	PA_d	10	F&P	multigene	non-cluster
OsIFCC031347	PA_d	10	C	multigene	non-cluster
OsIFCC031371	PA_d	10	F	multigene	non-cluster
OsIFCC031415	PA_d	10	C	multigene	non-cluster

OsIFCC031505	PA_d	10	C&F	multigene	non-cluster
OsIFCC031644	PA_d	10	C&F&P	multigene	non-cluster
OsIFCC031804	PA_d	10	F&P	multigene	non-cluster
OsIFCC044069	PA_d	10	C	multigene	non-cluster
OsIFCC029792	PA_d	10	unknown	multigene	non-cluster
OsIFCC029793	PA_d	10	unknown	multigene	non-cluster
OsIFCC030145	PA_d	10	unknown	multigene	non-cluster
OsIFCC030445	PA_d	10	unknown	multigene	non-cluster
OsIFCC030451	PA_d	10	unknown	multigene	non-cluster
OsIFCC030536	PA_d	10	unknown	multigene	non-cluster
OsIFCC030650	PA_d	10	unknown	multigene	non-cluster
OsIFCC030771	PA_d	10	unknown	multigene	non-cluster
OsIFCC030812	PA_d	10	unknown	multigene	non-cluster
OsIFCC031033	PA_d	10	unknown	multigene	non-cluster
OsIFCC031258	PA_d	10	unknown	multigene	non-cluster
OsIFCC031295	PA_d	10	unknown	multigene	non-cluster
OsIFCC031488	PA_d	10	unknown	multigene	non-cluster
OsIFCC031491	PA_d	10	unknown	multigene	non-cluster
OsIFCC044058	PA_d	10	unknown	multigene	non-cluster
OsIFCC044141	PA_d	10	unknown	multigene	non-cluster
OsIFCC044334	PA_d	10	unknown	multigene	non-cluster
OsIFCC029777	PA_d	10	unknown	multigene	non-cluster
OsIFCC030068	PA_d	10	unknown	multigene	non-cluster
OsIFCC030193	PA_d	10	C	multigene	non-cluster
OsIFCC029789	PA_d	10	C&F	multigene	non-cluster
OsIFCC030262	PA_d	10	C&F&P	multigene	non-cluster
OsIFCC030293	PA_d	10	C&F&P	multigene	non-cluster
OsIFCC030456	PA_d	10	C&F&P	multigene	non-cluster
OsIFCC030618	PA_d	10	C&F&P	multigene	non-cluster
OsIFCC030651	PA_d	10	C&F&P	multigene	non-cluster
OsIFCC031475	PA_d	10	C&F&P	multigene	non-cluster
OsIFCC044068	PA_d	10	C&F&P	multigene	non-cluster
OsIFCC044414	PA_d	10	C&F&P	multigene	non-cluster
OsIFCC044512	PA_d	10	C&F&P	multigene	non-cluster
OsIFCC031370	PA_d	10	C&F&P	multigene	cluster
OsIFCC031372	PA_d	10	C&F&P	multigene	cluster
OsIFCC029732	PA_d	10	C&F&P	multigene	non-cluster
OsIFCC031221	PA_d	10	C&F&P	multigene	non-cluster
OsIFCC030292	PA_d	10	C&P	multigene	non-cluster
OsIFCC030294	PA_d	10	C&P	multigene	non-cluster
OsIFCC030872	PA_d	10	C&P	multigene	non-cluster
OsIFCC044367	PA_d	10	C&P	multigene	non-cluster
OsIFCC044378	PA_d	10	C&P	multigene	non-cluster
OsIFCC044253	PA_d	10	C&P	multigene	non-cluster

OsIFCC030711	PA_d	10	F	multigene	non-cluster
OsIFCC029798	PA_d	10	F	multigene	non-cluster
OsIFCC029719	PA_d	10	F&P	multigene	non-cluster
OsIFCC030066	PA_d	10	F&P	multigene	non-cluster
OsIFCC030430	PA_d	10	F&P	multigene	non-cluster
OsIFCC030640	PA_d	10	F&P	multigene	non-cluster
OsIFCC030989	PA_d	10	F&P	multigene	non-cluster
OsIFCC031570	PA_d	10	F&P	multigene	non-cluster
OsIFCC044369	PA_d	10	F&P	multigene	non-cluster
OsIFCC030144	PA_d	10	P	multigene	non-cluster
OsIFCC030535	PA_d	10	P	multigene	non-cluster
OsIFCC044112	PA_d	10	P	multigene	non-cluster
**Genes in inserts of aligned contigs in Nipponbare**					
LOC_Os01g05930	PA	1	C&F&P	single	non-cluster
LOC_Os01g38360	PA	1	C&F&P	single	non-cluster
LOC_Os01g51370	PA	1	unknown	single	non-cluster
LOC_Os02g44090	PA	2	C	single	non-cluster
LOC_Os03g45280	PA	3	unknown	single	non-cluster
LOC_Os04g31090	PA	4	C&F&P	single	non-cluster
LOC_Os05g36980	PA	5	F&P	single	non-cluster
LOC_Os05g49330	PA	5	C&F&P	single	non-cluster
LOC_Os06g13950	PA	6	ND[c]	single	non-cluster
LOC_Os06g16790	PA	6	F&P	single	non-cluster
LOC_Os07g18930	PA	7	unknown	single	non-cluster
LOC_Os07g36050	PA	7	ND	multigene	cluster
LOC_Os07g41850	PA	7	P	single	non-cluster
LOC_Os07g41860	PA	7	C&F&P	single	non-cluster
LOC_Os08g30840	PA	8	C	single	non-cluster
LOC_Os10g36300	PA	10	unknown	single	non-cluster
LOC_Os10g36330	PA	10	C	single	non-cluster
LOC_Os11g13900	PA	11	unknown	multigene	non-cluster
LOC_Os11g13910	PA	11	C	multigene	non-cluster
LOC_Os12g34470	PA	12	P	single	non-cluster
LOC_Os01g04430	AL	1	F&P	multigene	cluster
LOC_Os01g05920	AL	1	unknown	multigene	non-cluster
LOC_Os01g06950	AL	1	P	multigene	cluster
LOC_Os01g10170	AL	1	C&F	multigene	non-cluster
LOC_Os01g10650	AL	1	ND[C]	multigene	non-cluster
LOC_Os01g12370	AL	1	C&F&P	multigene	non-cluster
LOC_Os01g35960	AL	1	unknown	multigene	non-cluster
LOC_Os01g43020	AL	1	C&F&P	single	non-cluster
LOC_Os01g67630	AL	1	C&F&P	multigene	cluster
LOC_Os02g34360	AL	2	unknown	multigene	cluster
LOC_Os03g18970	AL	3	unknown	multigene	non-cluster

LOC_Os03g19770	AL	3	C&F&P	multigene	non-cluster
LOC_Os03g47500	AL	3	P	multigene	non-cluster
LOC_Os04g04050	AL	4	P	multigene	cluster
LOC_Os04g04060	AL	4	C&F&P	multigene	non-cluster
LOC_Os04g25520	AL	4	unknown	multigene	non-cluster
LOC_Os04g25530	AL	4	unknown	multigene	non-cluster
LOC_Os04g38270	AL	4	unknown	single	non-cluster
LOC_Os04g53470	AL	4	C&F&P	multigene	cluster
LOC_Os04g53830	AL	4	C&F&P	multigene	cluster
LOC_Os04g53850	AL	4	unknown	multigene	cluster
LOC_Os05g06300	AL	5	F&P	multigene	non-cluster
LOC_Os05g19910	AL	5	unknown	single	non-cluster
LOC_Os05g19920	AL	5	unknown	single	non-cluster
LOC_Os05g28150	AL	5	F&P	multigene	non-cluster
LOC_Os06g07180	AL	6	P	single	non-cluster
LOC_Os06g07190	AL	6	unknown	multigene	non-cluster
LOC_Os06g07200	AL	6	P	single	non-cluster
LOC_Os06g07210	AL	6	F&P	multigene	non-cluster
LOC_Os06g09140	AL	6	F&P	multigene	non-cluster
LOC_Os06g16780	AL	6	unknown	multigene	non-cluster
LOC_Os06g21840	AL	6	C&F&P	multigene	non-cluster
LOC_Os06g24860	AL	6	unknown	multigene	non-cluster
LOC_Os06g39080	AL	6	C&F&P	multigene	cluster
LOC_Os07g07400	AL	7	C&F&P	multigene	cluster
LOC_Os07g07410	AL	7	F&P	single	non-cluster
LOC_Os07g07420	AL	7	F&P	single	non-cluster
LOC_Os07g18940	AL	7	unknown	multigene	non-cluster
LOC_Os07g25770	AL	7	C&F&P	multigene	non-cluster
LOC_Os07g45960	AL	7	F	multigene	non-cluster
LOC_Os07g49140	AL	7	C&F&P	single	non-cluster
LOC_Os07g49150	AL	7	C&F&P	multigene	non-cluster
LOC_Os08g30460	AL	8	F&P	multigene	non-cluster
LOC_Os08g41450	AL	8	unknown	multigene	non-cluster
LOC_Os08g44040	AL	8	C&F&P	multigene	cluster
LOC_Os09g12150	AL	9	unknown	multigene	non-cluster
LOC_Os09g23180	AL	9	C&F&P	multigene	non-cluster
LOC_Os10g11100	AL	10	C	multigene	non-cluster
LOC_Os10g23250	AL	10	unknown	multigene	non-cluster
LOC_Os10g24930	AL	10	C	multigene	non-cluster
LOC_Os10g35900	AL	10	unknown	multigene	non-cluster
LOC_Os10g36320	AL	10	F	multigene	non-cluster
LOC_Os11g34270	AL	11	C&F&P	single	non-cluster
LOC_Os12g07970	AL	12	C&P	multigene	non-cluster
LOC_Os12g15230	AL	12	C&P	multigene	non-cluster

LOC_Os12g27210	AL	12	C&F&P	multigene	non-cluster
LOC_Os01g03470	PA_d	1	P	multigene	non-cluster
LOC_Os01g08050	PA_d	1	P	multigene	non-cluster
LOC_Os01g22570	PA_d	1	P	multigene	non-cluster
LOC_Os01g43010	PA_d	1	P	multigene	non-cluster
LOC_Os01g54310	PA_d	1	C&F	multigene	non-cluster
LOC_Os01g64220	PA_d	1	F	multigene	non-cluster
LOC_Os02g13390	PA_d	2	ND	multigene	non-cluster
LOC_Os02g36820	PA_d	2	P	multigene	non-cluster
LOC_Os02g37310	PA_d	2	F	multigene	cluster
LOC_Os03g03490	PA_d	3	C&F&P	multigene	non-cluster
LOC_Os03g24770	PA_d	3	P	multigene	non-cluster
LOC_Os03g24810	PA_d	3	C&F	multigene	non-cluster
LOC_Os04g09730	PA_d	4	unknown	multigene	non-cluster
LOC_Os04g31100	PA_d	4	F	multigene	non-cluster
LOC_Os04g38280	PA_d	4	C&P	multigene	non-cluster
LOC_Os04g38290	PA_d	4	F	multigene	cluster
LOC_Os04g49280	PA_d	4	C&F&P	multigene	non-cluster
LOC_Os04g50050	PA_d	4	C&F&P	multigene	non-cluster
LOC_Os04g50840	PA_d	4	P	multigene	non-cluster
LOC_Os04g53840	PA_d	4	C	multigene	non-cluster
LOC_Os05g43330	PA_d	5	C&F&P	multigene	non-cluster
LOC_Os05g50530	PA_d	5	C&F&P	multigene	non-cluster
LOC_Os06g03100	PA_d	6	F	multigene	non-cluster
LOC_Os06g15240	PA_d	6	F	multigene	non-cluster
LOC_Os06g16770	PA_d	6	F&P	multigene	non-cluster
LOC_Os06g49360	PA_d	6	F&P	multigene	cluster
LOC_Os06g49380	PA_d	6	F&P	multigene	non-cluster
LOC_Os06g49410	PA_d	6	P	multigene	non-cluster
LOC_Os07g10180	PA_d	7	unknown	multigene	non-cluster
LOC_Os07g11550	PA_d	7	unknown	multigene	non-cluster
LOC_Os07g18950	PA_d	7	unknown	multigene	non-cluster
LOC_Os07g31680	PA_d	7	F&P	multigene	non-cluster
LOC_Os07g36060	PA_d	7	F&P	multigene	cluster
LOC_Os07g46470	PA_d	7	unknown	multigene	non-cluster
LOC_Os08g01540	PA_d	8	C&F&P	multigene	non-cluster
LOC_Os08g10150	PA_d	8	P	multigene	cluster
LOC_Os08g30690	PA_d	8	unknown	multigene	non-cluster
LOC_Os09g03150	PA_d	9	unknown	multigene	non-cluster
LOC_Os09g07210	PA_d	9	unknown	multigene	non-cluster
LOC_Os09g21720	PA_d	9	F	multigene	non-cluster
LOC_Os09g26010	PA_d	9	F	multigene	non-cluster
LOC_Os09g39130	PA_d	9	C&F&P	multigene	non-cluster
LOC_Os10g03890	PA_d	10	C	multigene	non-cluster

LOC_Os10g07060	PA_d	10	C	multigene	non-cluster
LOC_Os10g24950	PA_d	10	C&P	multigene	non-cluster
LOC_Os10g24960	PA_d	10	C&F&P	multigene	non-cluster
LOC_Os10g30230	PA_d	10	unknown	multigene	non-cluster
LOC_Os10g39700	PA_d	10	P	multigene	non-cluster
LOC_Os11g07640	PA_d	11	unknown	multigene	cluster
LOC_Os11g29600	PA_d	11	unknown	multigene	non-cluster
LOC_Os11g40010	PA_d	11	F	multigene	cluster
LOC_Os12g07920	PA_d	12	P	multigene	non-cluster
LOC_Os12g12140	PA_d	12	P	multigene	non-cluster
LOC_Os12g23150	PA_d	12	unknown	multigene	non-cluster
LOC_Os12g36710	PA_d	12	F&P	multigene	cluster
LOC_Os12g39770	PA_d	12	C&F&P	multigene	non-cluster
**Genes in inserts of aligned contigs in 93-11**					
OsIFCC000246	PA	1	F	single	non-cluster
OsIFCC000370	PA	1	C&F&P	single	non-cluster
OsIFCC000410	PA	1	unknown	single	non-cluster
OsIFCC001724	PA	1	F	single	non-cluster
OsIFCC006841	PA	3	C&F&P	single	non-cluster
OsIFCC006842	PA	3	unknown	single	non-cluster
OsIFCC008120	PA	2	C&F&P	single	non-cluster
OsIFCC009848	PA	6	unknown	single	non-cluster
OsIFCC010119	PA	6	F	single	non-cluster
OsIFCC011269	PA	6	unknown	single	non-cluster
OsIFCC011559	PA	6	C&F&P	single	non-cluster
OsIFCC011886	PA	6	F&P	single	non-cluster
OsIFCC011887	PA	6	F&P	single	non-cluster
OsIFCC012429	PA	2	unknown	single	non-cluster
OsIFCC013587	PA	2	F&P	single	non-cluster
OsIFCC013776	PA	2	C	single	non-cluster
OsIFCC014669	PA	2	F&P	single	non-cluster
OsIFCC015220	PA	2	C&F&P	single	non-cluster
OsIFCC016295	PA	4	C&F&P	single	non-cluster
OsIFCC016296	PA	4	C&F&P	single	non-cluster
OsIFCC016641	PA	4	unknown	single	non-cluster
OsIFCC017095	PA	4	F&P	single	non-cluster
OsIFCC018309	PA	4	unknown	single	non-cluster
OsIFCC019342	PA	4	F&P	single	non-cluster
OsIFCC019343	PA	4	F&P	single	non-cluster
OsIFCC019344	PA	4	C&F&P	single	non-cluster
OsIFCC019676	PA	5	F	single	non-cluster
OsIFCC022576	PA	7	unknown	single	non-cluster
OsIFCC023379	PA	7	C&F&P	single	non-cluster
OsIFCC023896	PA	7	unknown	single	non-cluster

OsIFCC024318	PA	7	C&F&P	single	non-cluster
OsIFCC024319	PA	7	C&F&P	single	non-cluster
OsIFCC025461	PA	8	C&F&P	single	non-cluster
OsIFCC026131	PA	8	unknown	single	non-cluster
OsIFCC027915	PA	3	C&F&P	single	non-cluster
OsIFCC028092	PA	9	unknown	single	non-cluster
OsIFCC029245	PA	9	C&F&P	single	non-cluster
OsIFCC030146	PA	10	C&F&P	multigene	non-cluster
OsIFCC031268	PA	10	C&F&P	single	non-cluster
OsIFCC031269	PA	10	F	single	non-cluster
OsIFCC032306	PA	12	F&P	single	non-cluster
OsIFCC032860	PA	12	unknown	single	non-cluster
OsIFCC033150	PA	12	F&P	single	non-cluster
OsIFCC034369	PA	11	P	single	non-cluster
OsIFCC034431	PA	11	C&P	single	non-cluster
OsIFCC034506	PA	11	unknown	single	non-cluster
OsIFCC034565	PA	11	C	single	non-cluster
OsIFCC036060	PA	11	C	single	non-cluster
OsIFCC037848	PA	3	C&F&P	single	non-cluster
OsIFCC038863	PA	6	P	single	non-cluster
OsIFCC040675	PA	4	P	single	non-cluster
OsIFCC041264	PA	5	unknown	single	non-cluster
OsIFCC042890	PA	8	C&F&P	single	non-cluster
OsIFCC043050	PA	8	C&P	single	non-cluster
OsIFCC043185	PA	8	C&F&P	single	non-cluster
OsIFCC044928	PA	12	unknown	single	non-cluster
OsIFCC045383	PA	11	C	single	non-cluster
OsIFSC046432	PA	NF[b]	P	single	non-cluster
OsIFSC046483	PA	NF	F&P	single	non-cluster
OsIFSC046484	PA	NF	unknown	single	non-cluster
OsIFSC046500	PA	NF	F	single	non-cluster
OsIFSC048165	PA	NF	unknown	single	non-cluster
OsIFCC002147	AL	1	P	single	non-cluster
OsIFCC002148	AL	1	unknown	multigene	non-cluster
OsIFCC003001	AL	1	P	single	non-cluster
OsIFCC003352	AL	1	C&F	single	non-cluster
OsIFCC003829	AL	1	F&P	single	non-cluster
OsIFCC003830	AL	1	unknown	single	non-cluster
OsIFCC004057	AL	1	unknown	single	non-cluster
OsIFCC005469	AL	3	F	multigene	non-cluster
OsIFCC005470	AL	3	unknown	multigene	non-cluster
OsIFCC007038	AL	3	C	single	non-cluster
OsIFCC007699	AL	3	F&P	single	non-cluster
OsIFCC007700	AL	3	P	multigene	non-cluster

OsIFCC007701	AL	3	F	multigene	non-cluster
OsIFCC008104	AL	3	unknown	single	non-cluster
OsIFCC008370	AL	3	C&F&P	single	non-cluster
OsIFCC008371	AL	3	F&P	multigene	cluster
OsIFCC008820	AL	3	P	single	non-cluster
OsIFCC008821	AL	3	C&F&P	single	non-cluster
OsIFCC008822	AL	3	C&F&P	multigene	non-cluster
OsIFCC008823	AL	3	C&F&P	single	non-cluster
OsIFCC009799	AL	6	F&P	single	non-cluster
OsIFCC010424	AL	6	C&F&P	single	non-cluster
OsIFCC010875	AL	6	unknown	single	non-cluster
OsIFCC011402	AL	6	P	single	non-cluster
OsIFCC011404	AL	6	C&F&P	single	non-cluster
OsIFCC011405	AL	6	C&F&P	single	non-cluster
OsIFCC012002	AL	6	F&P	single	non-cluster
OsIFCC012003	AL	6	F&P	single	non-cluster
OsIFCC012098	AL	6	P	single	non-cluster
OsIFCC012911	AL	2	F	single	non-cluster
OsIFCC013393	AL	2	unknown	single	non-cluster
OsIFCC016608	AL	4	C	multigene	non-cluster
OsIFCC016640	AL	4	unknown	single	non-cluster
OsIFCC016694	AL	4	C&F&P	single	non-cluster
OsIFCC018307	AL	4	P	single	non-cluster
OsIFCC018475	AL	4	unknown	single	non-cluster
OsIFCC018611	AL	4	C&F&P	multigene	non-cluster
OsIFCC018613	AL	4	C&F&P	multigene	non-cluster
OsIFCC018803	AL	4	C	multigene	cluster
OsIFCC019374	AL	5	unknown	single	non-cluster
OsIFCC019844	AL	5	C&F	single	non-cluster
OsIFCC020501	AL	5	unknown	single	non-cluster
OsIFCC020584	AL	5	C&F&P	single	non-cluster
OsIFCC021616	AL	5	C	single	non-cluster
OsIFCC022660	AL	7	F&P	single	non-cluster
OsIFCC023297	AL	7	unknown	single	non-cluster
OsIFCC023478	AL	7	C&F&P	single	non-cluster
OsIFCC023479	AL	7	unknown	single	non-cluster
OsIFCC023895	AL	7	unknown	multigene	non-cluster
OsIFCC024353	AL	7	C&F&P	single	non-cluster
OsIFCC024904	AL	7	F	single	non-cluster
OsIFCC024905	AL	7	F&P	single	non-cluster
OsIFCC025282	AL	8	F	multigene	cluster
OsIFCC025283	AL	8	C&F	multigene	cluster
OsIFCC026695	AL	8	F&P	single	non-cluster
OsIFCC026756	AL	8	F&P	single	non-cluster

OsIFCC026783	AL	8	F	multigene	non-cluster
OsIFCC026784	AL	8	unknown	multigene	cluster
OsIFCC026811	AL	8	unknown	single	non-cluster
OsIFCC027367	AL	8	C&F&P	multigene	cluster
OsIFCC027577	AL	8	C&P	single	non-cluster
OsIFCC027578	AL	8	F	single	non-cluster
OsIFCC027579	AL	8	C&F&P	single	non-cluster
OsIFCC027580	AL	8	C&P	multigene	non-cluster
OsIFCC028035	AL	9	unknown	multigene	non-cluster
OsIFCC028419	AL	9	C&F&P	single	non-cluster
OsIFCC028481	AL	9	C&F&P	single	non-cluster
OsIFCC029006	AL	9	unknown	multigene	cluster
OsIFCC029299	AL	9	F&P	single	non-cluster
OsIFCC029576	AL	9	C&F&P	single	non-cluster
OsIFCC030488	AL	10	unknown	multigene	non-cluster
OsIFCC030708	AL	10	C&P	multigene	non-cluster
OsIFCC032513	AL	12	C&F&P	single	non-cluster
OsIFCC032719	AL	12	C&P	multigene	non-cluster
OsIFCC033082	AL	12	F&P	single	non-cluster
OsIFCC033083	AL	12	F&P	single	non-cluster
OsIFCC033284	AL	12	C&F	multigene	non-cluster
OsIFCC033359	AL	12	unknown	single	non-cluster
OsIFCC033744	AL	12	C&F	single	non-cluster
OsIFCC034370	AL	11	F&P	single	non-cluster
OsIFCC034566	AL	11	C&F&P	multigene	non-cluster
OsIFCC034678	AL	11	unknown	single	non-cluster
OsIFCC036488	AL	1	unknown	multigene	cluster
OsIFCC036519	AL	1	F&P	multigene	non-cluster
OsIFCC038684	AL	6	unknown	single	non-cluster
OsIFCC039658	AL	2	unknown	multigene	non-cluster
OsIFCC040126	AL	2	C&F&P	single	non-cluster
OsIFCC040719	AL	4	unknown	multigene	non-cluster
OsIFCC041294	AL	5	P	single	non-cluster
OsIFCC041339	AL	5	C&P	multigene	non-cluster
OsIFCC042030	AL	7	unknown	multigene	non-cluster
OsIFCC042031	AL	7	unknown	single	non-cluster
OsIFCC042808	AL	8	F	multigene	cluster
OsIFCC042809	AL	8	C&F&P	single	non-cluster
OsIFCC042888	AL	8	C&F&P	single	non-cluster
OsIFCC043590	AL	9	P	multigene	non-cluster
OsIFCC044679	AL	12	unknown	multigene	non-cluster
OsIFCC044984	AL	12	C&F&P	multigene	non-cluster
OsIFCC045106	AL	12	F&P	multigene	non-cluster
OsIFSC048166	AL	NF	C&P	single	non-cluster

OsIFCC001432	PA_d	1	P	multigene	cluster
OsIFCC006341	PA_d	3	F&P	multigene	non-cluster
OsIFCC010118	PA_d	6	unknown	multigene	non-cluster
OsIFCC011403	PA_d	6	F&P	multigene	non-cluster
OsIFCC012906	PA_d	2	F	multigene	non-cluster
OsIFCC012910	PA_d	2	unknown	multigene	non-cluster
OsIFCC015356	PA_d	2	unknown	multigene	non-cluster
OsIFCC016225	PA_d	4	C	multigene	non-cluster
OsIFCC016294	PA_d	4	unknown	multigene	non-cluster
OsIFCC017282	PA_d	4	F&P	multigene	non-cluster
OsIFCC017650	PA_d	4	C&F&P	multigene	non-cluster
OsIFCC018310	PA_d	4	unknown	multigene	cluster
OsIFCC018311	PA_d	4	unknown	multigene	cluster
OsIFCC018314	PA_d	4	unknown	multigene	cluster
OsIFCC019950	PA_d	5	F	multigene	non-cluster
OsIFCC022752	PA_d	7	C&F&P	multigene	non-cluster
OsIFCC022753	PA_d	7	C&F&P	multigene	non-cluster
OsIFCC023891	PA_d	7	unknown	multigene	non-cluster
OsIFCC024322	PA_d	7	F	multigene	non-cluster
OsIFCC025333	PA_d	8	F	multigene	non-cluster
OsIFCC025460	PA_d	8	C&F&P	multigene	non-cluster
OsIFCC030074	PA_d	10	C&F&P	multigene	non-cluster
OsIFCC030144	PA_d	10	P	multigene	non-cluster
OsIFCC030145	PA_d	10	unknown	multigene	non-cluster
OsIFCC031257	PA_d	10	C&F	multigene	non-cluster
OsIFCC032899	PA_d	12	C&F&P	multigene	non-cluster
OsIFCC034368	PA_d	11	C&F	multigene	non-cluster
OsIFCC034371	PA_d	11	C&F&P	multigene	non-cluster
OsIFCC034372	PA_d	11	F&P	multigene	non-cluster
OsIFCC034503	PA_d	11	F	multigene	non-cluster
OsIFCC034577	PA_d	11	unknown	multigene	non-cluster
OsIFCC036035	PA_d	11	unknown	multigene	non-cluster
OsIFCC036518	PA_d	1	P	multigene	cluster
OsIFCC038440	PA_d	3	unknown	multigene	cluster
OsIFSC046501	PA_d	NF	F&P	multigene	non-cluster
**Genes in *R*-gene family in Nipponbare**					
AC135190a	PA	11	*R*-gene	single	cluster
AC135497b	PA	11	*R*-gene	single	cluster
AP005257	PA	7	*R*-gene	single	cluster
CC002584	PA	11	*R*-gene	single	cluster
CC005649	PA	2	*R*-gene	single	cluster
CC019339	PA	6	*R*-gene	single	cluster
CC019340	PA	6	*R*-gene	single	cluster
CC021577	PA	8	*R*-gene	single	cluster

CC021945	PA	11	*R*-gene	single	cluster
CC022580	PA	8	*R*-gene	single	cluster
CC023500	PA	8	*R*-gene	single	cluster
CC023501	PA	8	*R*-gene	single	cluster
CC025363	PA	7	*R*-gene	single	cluster
CC025364	PA	7	*R*-gene	single	cluster
CC027095	PA	11	*R*-gene	single	cluster
CC027099	PA	11	*R*-gene	single	cluster
CC027114	PA	11	*R*-gene	single	cluster
CC027129	PA	11	*R*-gene	single	cluster
CC027318	PA	11	*R*-gene	single	cluster
CC028567	PA	11	*R*-gene	single	cluster
CC031194	PA	12	*R*-gene	single	cluster
CC038058	PA	5	*R*-gene	single	cluster
CC040847	PA	7	*R*-gene	single	cluster
CC041278	PA	11	*R*-gene	single	cluster
CC041574	PA	11	*R*-gene	single	cluster
CC042530	PA	12	*R*-gene	single	cluster
AC114011a	PA	11	*R*-gene	single	non-cluster
AC135957	PA	11	*R*-gene	single	non-cluster
AP004555a	PA	8	*R*-gene	single	non-cluster
CC001216	PA	1	*R*-gene	single	non-cluster
CC005555	PA	2	*R*-gene	single	non-cluster
CC005653	PA	2	*R*-gene	single	non-cluster
CC015953	PA	4	*R*-gene	single	non-cluster
CC019293	PA	6	*R*-gene	single	non-cluster
CC022665	PA	8	*R*-gene	single	non-cluster
CC024939	PA	7	*R*-gene	single	non-cluster
CC027323	PA	11	*R*-gene	single	non-cluster
CC032900	PA	10	*R*-gene	single	non-cluster
CC036156	PA	2	*R*-gene	single	non-cluster
CC038372	PA	4	*R*-gene	single	non-cluster
CC041795	PA	9	*R*-gene	single	non-cluster
CC042517	PA	12	*R*-gene	single	non-cluster
CC042758	PA	10	*R*-gene	single	non-cluster
AL607003	PA_d	4	*R*-gene	multigene	cluster
AL713909b	PA_d	12	*R*-gene	multigene	cluster
AP003368d	PA_d	1	*R*-gene	multigene	cluster
AP005501d	PA_d	8	*R*-gene	multigene	cluster
CC000083	PA_d	1	*R*-gene	multigene	cluster
CC012075	PA_d	3	*R*-gene	multigene	cluster
CC013868	PA_d	2	*R*-gene	multigene	cluster
CC019239	PA_d	6	*R*-gene	multigene	cluster
CC020989	PA_d	5	*R*-gene	multigene	cluster

CC020992	PA_d	6	*R*-gene	multigene	cluster
CC026082	PA_d	7	*R*-gene	multigene	cluster
CC027124	PA_d	11	*R*-gene	multigene	cluster
CC028187	PA_d	11	*R*-gene	multigene	cluster
CC028189	PA_d	9	*R*-gene	multigene	cluster
CC028270	PA_d	11	*R*-gene	multigene	cluster
CC028539	PA_d	11	*R*-gene	multigene	cluster
CC029261	PA_d	9	*R*-gene	multigene	cluster
CC032223	PA_d	12	*R*-gene	multigene	cluster
CC032225	PA_d	12	*R*-gene	multigene	non-cluster
CC037657	PA_d	3	*R*-gene	multigene	cluster
CC041286	PA_d	5	*R*-gene	multigene	cluster
**Genes in *R*-gene family in 93-11**					
AAAA01000862_6	PA	NF	*R*-gene	single	ND
AAAA01000924_4	PA	7	*R*-gene	single	cluster
AAAA01001274_5	PA	1	*R*-gene	single	cluster
AAAA01001660_1	PA	7	*R*-gene	single	non-cluster
AAAA01001715_5	PA	10	*R*-gene	single	non-cluster
AAAA01001765_1	PA	2	*R*-gene	single	cluster
AAAA01001765_5	PA	2	*R*-gene	single	cluster
AAAA01001803_4	PA	5	*R*-gene	single	non-cluster
AAAA01003047_1	PA	4	*R*-gene	single	cluster
AAAA01003554_4	PA	2	*R*-gene	single	non-cluster
AAAA01003777_3	PA	8	*R*-gene	single	non-cluster
AAAA01004171_1	PA	NF	*R*-gene	single	ND
AAAA01004234_2	PA	NF	*R*-gene	single	ND
AAAA01004624_1	PA	11	*R*-gene	single	cluster
AAAA01004640_3	PA	11	*R*-gene	single	cluster
AAAA01005128_1	PA	7	*R*-gene	single	cluster
AAAA01005471_1	PA	8	*R*-gene	single	cluster
AAAA01005607_1	PA	3	*R*-gene	single	non-cluster
AAAA01005808_1	PA	12	*R*-gene	single	cluster
AAAA01006940_2	PA	1	*R*-gene	single	cluster
AAAA01009049_1	PA	6	*R*-gene	single	cluster
AAAA01009621_2	PA	NF	*R*-gene	single	ND
AAAA01009698_1	PA	11	*R*-gene	single	cluster
AAAA01009793_1	PA	7	*R*-gene	single	cluster
AAAA01010257_1	PA	6	*R*-gene	single	non-cluster
AAAA01010541_2	PA	8	*R*-gene	single	non-cluster
AAAA01010924_1	PA	9	*R*-gene	single	non-cluster
AAAA01010962_1	PA	11	*R*-gene	single	cluster
AAAA01011627_1	PA	11	*R*-gene	single	cluster
AAAA01013177_1	PA	12	*R*-gene	single	non-cluster
AAAA01013445_1	PA	5	*R*-gene	single	cluster

AAAA01014728_1	PA	7	*R*-gene	single	cluster
AAAA01016026_1	PA	6	*R*-gene	single	non-cluster
OsIFCC002166	PA	4	*R*-gene	single	cluster
OsIFCC006484	PA	3	*R*-gene	single	non-cluster
OsIFCC007375	PA	3	*R*-gene	single	cluster
OsIFCC007377	PA	3	*R*-gene	single	cluster
OsIFCC010265	PA	6	*R*-gene	single	cluster
OsIFCC011502	PA	2	*R*-gene	single	non-cluster
OsIFCC012619	PA	2	*R*-gene	single	non-cluster
OsIFCC016966	PA	4	*R*-gene	single	non-cluster
OsIFCC023445	PA	7	*R*-gene	single	cluster
OsIFCC033126	PA	12	*R*-gene	single	non-cluster
OsIFCC033585	PA	12	*R*-gene	single	cluster
OsIFCC033812	PA	12	*R*-gene	single	non-cluster
OsIFCC034390	PA	11	*R*-gene	single	non-cluster
OsIFCC034501	PA	11	*R*-gene	single	non-cluster
OsIFCC034563	PA	11	*R*-gene	single	cluster
OsIFCC035221	PA	11	*R*-gene	single	cluster
OsIFCC035693	PA	11	*R*-gene	single	cluster
OsIFCC035753	PA	11	*R*-gene	single	cluster
OsIFCC035768	PA	11	*R*-gene	single	non-cluster
OsIFCC035797	PA	11	*R*-gene	single	cluster
OsIFCC035800	PA	11	*R*-gene	single	non-cluster
OsIFCC036063	PA	11	*R*-gene	single	non-cluster
OsIFSC046148	PA	NF	*R*-gene	single	ND
OsIFSC046157	PA	11	*R*-gene	single	cluster
OsIFSC046157	PA	11	*R*-gene	single	cluster
OsIFSC046867	PA	ChrUn	*R*-gene	single	ND
OsIFSC046976	PA	ChrUn	*R*-gene	single	ND
OsIFSC047074	PA	ChrUn	*R*-gene	single	ND
OsIFSC047502	PA	ChrUn	*R*-gene	single	ND
AAAA02012175	AL	4	*R*-gene	single	non-cluster
AAAA02011393	AL	3	*R*-gene	multigene	cluster
AAAA02006137	AL	2	*R*-gene	single	non-cluster
AAAA02005670	AL	2	*R*-gene	single	non-cluster
AAAA02011796	AL	4	*R*-gene	single	non-cluster
AAAA02008304	AL	3	*R*-gene	single	non-cluster
AAAA02029432	AL	10	*R*-gene	single	cluster
AAAA01009531_2	AL	10	*R*-gene	multigene	cluster
AAAA02012853	AL	4	*R*-gene	single	cluster
AAAA02028520	AL	10	*R*-gene	single	non-cluster
AAAA02015947_1	AL	5	*R*-gene	multigene	cluster
AAAA02015947_2	AL	5	*R*-gene	single	cluster
AAAA02002233	AL	1	*R*-gene	single	cluster

AAAA02019574	AL	6	*R*-gene	single	non-cluster
AAAA02023008	AL	8	*R*-gene	single	non-cluster
AAAA02027233	AL	9	*R*-gene	single	cluster
AAAA02031031	AL	11	*R*-gene	single	cluster
AAAA02031042	AL	11	*R*-gene	single	cluster
AAAA02031967	AL	11	*R*-gene	single	cluster
AAAA02033127_2	AL	11	*R*-gene	single	cluster
AAAA02034638	AL	12	*R*-gene	single	non-cluster
AAAA02034655	AL	12	*R*-gene	single	cluster
AAAA02035717	AL	ChrUn	*R*-gene	single	cluster
OsIFCC005137	AL	3	*R*-gene	single	cluster
OsIFCC005138	AL	3	*R*-gene	multigene	cluster
OsIFCC005153	AL	3	*R*-gene	single	cluster
OsIFCC009427	AL	6	*R*-gene	single	cluster
OsIFCC009428	AL	6	*R*-gene	multigene	cluster
OsIFCC010261	AL	6	*R*-gene	single	cluster
OsIFCC011501	AL	6	*R*-gene	multigene	non-cluster
OsIFCC011506	AL	6	*R*-gene	multigene	cluster
OsIFCC012956	AL	2	*R*-gene	single	cluster
OsIFCC016816	AL	4	*R*-gene	multigene	cluster
OsIFCC017218	AL	4	*R*-gene	multigene	cluster
OsIFCC019876	AL	5	*R*-gene	single	non-cluster
OsIFCC019892	AL	5	*R*-gene	multigene	cluster
OsIFCC019896	AL	5	*R*-gene	single	cluster
OsIFCC020204	AL	5	*R*-gene	multigene	cluster
OsIFCC020233	AL	5	*R*-gene	multigene	cluster
OsIFCC020397	AL	5	*R*-gene	single	cluster
OsIFCC020845	AL	5	*R*-gene	single	cluster
OsIFCC023859	AL	7	*R*-gene	single	cluster
OsIFCC025328	AL	8	*R*-gene	multigene	cluster
OsIFCC026178	AL	8	*R*-gene	single	non-cluster
OsIFCC027453	AL	8	*R*-gene	single	cluster
OsIFCC029246	AL	9	*R*-gene	multigene	cluster
OsIFCC029808	AL	10	*R*-gene	single	cluster
OsIFCC029848	AL	10	*R*-gene	single	cluster
OsIFCC034567	AL	11	*R*-gene	multigene	cluster
OsIFCC035230	AL	11	*R*-gene	single	cluster
OsIFCC035961	AL	11	*R*-gene	single	non-cluster
OsIFCC036006	AL	11	*R*-gene	multigene	cluster
OsIFCC036043	AL	11	*R*-gene	multigene	cluster
OsIFCC036049	AL	11	*R*-gene	multigene	cluster
OsIFCC036537	AL	1	*R*-gene	single	non-cluster
OsIFCC041626	AL	5	*R*-gene	multigene	cluster
OsIFCC042075	AL	7	*R*-gene	single	cluster

OsIFCC043691	AL	9	*R*-gene	single	cluster
AAAA02021242	PA_d	7	*R*-gene	multigene	cluster
AAAA02005199	PA_d	2	*R*-gene	multigene	cluster
AAAA02034526	PA_d	12	*R*-gene	multigene	cluster
AAAA02014792	PA_d	4	*R*-gene	multigene	cluster
AAAA02015914	PA_d	5	*R*-gene	multigene	cluster
AAAA02033186	PA_d	11	*R*-gene	multigene	cluster
AAAA02034995	PA_d	12	*R*-gene	multigene	cluster
AAAA01016632_1	PA_d	8	*R*-gene	multigene	cluster
OsIFCC009553	PA_d	6	*R*-gene	multigene	cluster
OsIFCC020218	PA_d	5	*R*-gene	multigene	non-cluster
OsIFCC020874	PA_d	5	*R*-gene	multigene	non-cluster
OsIFCC029249	PA_d	9	*R*-gene	multigene	cluster
OsIFCC033204	PA_d	12	*R*-gene	multigene	cluster
OsIFCC035222	PA_d	11	*R*-gene	multigene	cluster
OsIFCC035984	PA_d	11	*R*-gene	multigene	cluster
OsIFCC036010	PA_d	11	*R*-gene	multigene	cluster
OsIFCC036026	PA_d	11	*R*-gene	multigene	non-cluster
OsIFCC039636	PA_d	2	*R*-gene	multigene	cluster
OsIFCC042307	PA_d	7	*R*-gene	multigene	non-cluster
OsIFCC045762	PA_d	11	*R*-gene	multigene	cluster
OsIFSC046529	PA_d	11	*R*-gene	multigene	cluster
**Genes in *RLK* gene family in Nipponbare**					
3778.m00100	PA	2	Kinase	single	cluster
5928.m00110	PA	9	Kinase	single	non-cluster
6247.m00042	PA	2	Kinase	single	non-cluster
7014.m00090	PA	7	Kinase	single	non-cluster
1947.m00139	AL	6	Kinase	single	cluster
1954.m00189	AL	6	Kinase	single	cluster
2493.m00131	AL	2	Kinase	multigene	cluster
2493.m00133	AL	2	Kinase	multigene	cluster
2507.m00094	AL	2	Kinase	multigene	cluster
2712.m00179	AL	1	Kinase	multigene	cluster
2872.m00058	AL	1	Kinase	multigene	non-cluster
2942.m00117	AL	2	Kinase	single	non-cluster
2973.m00130	AL	5	Kinase	single	non-cluster
3181.m00133	AL	10	Kinase	single	cluster
3778.m00106	AL	2	Kinase	single	non-cluster
3841.m00143	AL	2	Kinase	multigene	cluster
3852.m00140	AL	2	Kinase	single	cluster
3852.m00143	AL	2	Kinase	multigene	cluster
3852.m00146	AL	2	Kinase	multigene	cluster
4279.m00143	AL	2	Kinase	multigene	cluster
4440.m00144	AL	1	Kinase	single	cluster

5104.m00185	AL	6	Kinase	multigene	cluster
5168.m00126	AL	8	Kinase	multigene	non-cluster
5500.m00225	AL	4	Kinase	multigene	non-cluster
5531.m00139	AL	4	Kinase	single	non-cluster
5928.m00133	AL	9	Kinase	single	non-cluster
6103.m00140	AL	10	Kinase	single	non-cluster
6242.m00122	AL	9	Kinase	single	cluster
6324.m00130	AL	11	Kinase	multigene	cluster
6324.m00132	AL	11	Kinase	single	cluster
6324.m00133	AL	11	Kinase	multigene	cluster
6324.m00134	AL	11	Kinase	multigene	cluster
6324.m00144	AL	11	Kinase	multigene	cluster
6324.m00146	AL	11	Kinase	multigene	cluster
6498.m00140	AL	6	Kinase	single	non-cluster
6522.m00161	AL	11	Kinase	single	cluster
6574.m00049	AL	6	Kinase	single	cluster
6592.m00198	AL	6	Kinase	multigene	cluster
6592.m00214	AL	6	Kinase	multigene	cluster
6592.m00220	AL	6	Kinase	multigene	cluster
6850.m00166	AL	11	Kinase	multigene	cluster
6850.m00171	AL	11	Kinase	multigene	cluster
6850.m00174	AL	11	Kinase	multigene	cluster
6850.m00176	AL	11	Kinase	multigene	cluster
6927.m00147	AL	8	Kinase	single	non-cluster
6958.m00101	AL	8	Kinase	multigene	non-cluster
6983.m00134	AL	1	Kinase	single	cluster
7342.m00113	AL	11	Kinase	multigene	cluster
7396.m00099	AL	11	Kinase	single	non-cluster
8186.m00129	AL	4	Kinase	single	non-cluster
8186.m00131	AL	4	Kinase	multigene	cluster
8186.m00132	AL	4	Kinase	multigene	cluster
8210.m00132	AL	4	Kinase	single	non-cluster
8221.m00128	AL	2	Kinase	multigene	cluster
8288.m00116	AL	4	Kinase	multigene	cluster
**Genes in *RLK* gene family in 93-11**					
CC009562	PA	6	Kinase	single	cluster
CC009564	PA	6	Kinase	single	cluster
CC011260	PA	6	Kinase	single	cluster
CC014664	PA	2	Kinase	multigene	cluster
CC014665	PA	2	Kinase	multigene	cluster
CC014690	PA	2	Kinase	single	non-cluster
CC017094	PA	4	Kinase	single	cluster
CC021621	PA	5	Kinase	single	cluster
CC021622	PA	5	Kinase	single	cluster

CC033150	PA	12	Kinase	single	non-cluster
CC035782	PA	11	Kinase	multigene	non-cluster
CC035790	PA	11	Kinase	multigene	non-cluster
CC036052	PA	11	Kinase	single	cluster
**Genes in *Myb* gene family in 93-11**					
CC020386	PA	5	transcription factor	single	ND
CC028541	PA	9	transcription factor	single	ND
CC032332	PA	12	transcription factor	single	ND
AAAA02030895	AL	11	transcription factor	single	non-cluster
**Genes in *MADS* gene family in 93-11**					
CC001468	PA	1	transcription factor	single	ND
CC008363	PA	3	transcription factor	single	ND
CC016421	PA	4	transcription factor	single	ND
AAAA02034103	AL	12	transcription factor	multigene	non-cluster

[a] GO Categories, P: biological_process; C: cellular_component; F: molecular_function.

[b] Contigs that included in the short-gun sequence database but cannot be found in the chromosome database of 93-11.

[c] The GO Category of the gene has not been determined.
