# Supplementary material for: Highly asymmetric rice genomes
Source: BMC Genomics. 2007 Jun 8;8:154. doi: 10.1186/1471-2164-8-154 (PMC1914357; doi:10.1186/1471-2164-8-154)
Supplement: Additional file 2 — Table S2–S4 [file 1471-2164-8-154-S2.pdf]

Table S2: The total percentage of TE homologous sequences in symmetric (allelic) and asymmetric (PA, PA_d and AL) genes on chromosome 10.

TE%	Genes in Nipponbare				Genes in 93-11			
(length) [a]	Allelic	PA	PA_d	AL	Allelic	PA	PA_d	AL
0	9(8%) [b]	8(14%)	5(10%)	6(9%)	4(3%)	2(3%)	4(6%)	1(2%)
0-5%	44(37%)	9(16%)	15(31%)	15(23%)	57(45%)	33(49%)	29(40%)	14(30%)
5%-10%	27(23%)	22(39%)	14(29%)	22(34%)	35(28%)	17(25%)	13(18%)	13(28%)
10%-20%	28(23%)	12(21%)	7(14%)	14(22%)	22(17%)	9(13%)	21(29%)	10(22%)
>20%	12(10%)	5(9%)	8(16%)	7(11%)	8(6%)	7(10%)	5(7%)	8(17%)
Total genes	120	56	49	64	126	68	72	46

[a] The proportion of TE-related fragments (the total length of all small pieces of TE homologous sequences contained in a gene divided by the length of this gene).

[b] The first number is the amount of the genes within each range with its corresponding percentage in brackets.

Table S3: The sequence sizes and numbers of non-alignable regions (insertions) in Nipponbare and 93-11 on chromosome 10.

Nip. insertion length	93-11 insertion length					
	<0bp	0-300bp	300bp-1kb	1kb-5kb	5kb-10kb	>10kb
<0bp	/	6	151	**183**	**56**	**61**
0-300bp	11	4	34	**81**	**19**	**25**
300bp-1kb	151	34	18	**47**	**16**	**13**
1kb-5kb	**171**	**31**	**16**	52	22	25
5kb-10kb	**64**	**11**	**8**	20	15	16
>10kb	**59**	**5**	**9**	23	18	60

Note. The bolded numbers represent the insertions and the others (>1 kb) stand for the non-alignable regions which were used in this study.

Table S4: Primer information for the amplification of gene fragments in Table 3.

NO.	Gene	Length	Reverse primer (5' to 3')
1	OsIFCC009208	832bp	GACAATTCAGGGAGGTTACGG
			GAGCGAGCAGAAGCAGTGG
2	OsIFCC035754	828bp	AAGGTGAAAGTGTGGAGGAGAT
			CAGAGGTTGGGGATTCGTAG
3	OsIFSC047991	1078bp	CATGCGACGAAGGTTGACTA
			GAGGGAGTGCTTTGGTGTCA
4	OsIFCC019895	859bp	GAACAAGAAGGGGCGGTGAA
			GCAGGTGTCGCAATGGTGAA
5	OsIFCC035751	831bp	TTACGAGATTCCAAGAGACCAA
			TCCACCAGAGTTTCCATCAAG
6	OsIFCC035229	1013bp	GTTTTGCGAGTATTGGATGTTG
			ATTGGATTGTTGGGATTATTGA
7	OsIFCC043050	1001bp	ACAACAAGAGCCCCCATAGC
			CGTCTCCAACATCGGCATAG
8	OsIFCC045542	986bp	CCATTCTCTGCCTTCCTCTACC
			TGCTTTTCAGTGTCAACCTCTC
9	OsIFCC023444	917bp	GGATGCTTGGGGTCAGATTT
			CAGTGCTCATTTCGTCATTGT
10	OsIFCC026523	1062bp	CCCTTCTTTGGTGGCATTCA
			GGGTGGTTGGGATGAGTGCT
11	OsIFSC046156	782bp	AGGGAGGAGCCATCTTCTATT
			TTCCGCCTTCACCTAATGTC
12	Os12g31620	708bp	CCTCCGCCACCTGTCTGTAA
			CGTGCCAAGCCCCTAATGAA
13	Os11g45330	1089bp	GTTGATTTGTGCCGAAGAGA
			ATGCCACGGAGGTTTATTAGA
14	Os06g16790	928bp	GAGCTTAGGCGGGAGAAATCAC
			CACCTTTCCAGCCATCCTCTAC
15	Os12g09730	1099bp	ACGAGCAGCGACGAATCAAG
			GCGAGGAGGGCACACAGAA
16	Os10g04670	1014bp	GTGAGTTGGCGATTGACCTG
			GATTAGGCAAGTCCCGCAGT
17	Os11g11550	844bp	TTGTTTCCTCGCCTGATGGT
			AAACGCCAGAGCAGCATCTTA
18	Os12g28250	1004bp	GGCACCTACGCTCCTTGAT
			TGGCTGTCACTTCAACCTCT
19	Os11g15670	1029bp	TACATTGCCGCTACTGGGAG
			AAAGGCATTCTTGGGCAGAG
20	Os01g20720	1077bp	GCGGTGTTCCTCTGACGATA
			GTTCTGCCACTCCTGCTGTC
21	Os12g18360	992bp	AATGCACATGATACAACACCACT

			AGCCACGGCAGAGAAAGAAAG
22	Os04g41370	942bp	AATCACAGACATCCCAGAAGAA
			GTGCCATCATCCCCATAGAA
23	Os07g29820	1061bp	ACAGATAGGTAGAGCCGAGAA
			ACAAGTGGGCAGCAACAAA
24	Os02g25900	970bp	AAAGCAGCAGATGAGCCTAAG
			AGTGGCCGATGATGATGAAG
25	OsIFCC040819	561bp	GGGTGGAGTGGGCAAGACAAC
			TTGAACGACAGCAGGCGACC
26	Os12g37270	921bp	AGCAACTGGAAGGGAGATAAG
			AACATCGTGCTCCAATAGCCT
27	Os12g03750	1068bp	GGGAGGCAGTTATTTGATGAG
			CTGGAAGAATTTGATGGTGAGT
28	Os01g57270	899bp	CAAACAACCTGGAAACACAACT
			AACTCTGGAACAACGAAACCT
29	Os05g16180	904bp	GCAGATGGTGAGTGGTGTAGTT
			GCAGGTATCTTGGGAGTTGTT
30	Os08g10430	1060bp	AGCAAGTGCAAGGAGGATAA
			TGGAGCTTGGGCATTACAC
31	Os01g05490	579bp	TCAGCCCACCTTATGT
			CACCACCGACGAGAAA
32	Os03g61120	207bp	AGGCTCGTGGTTGTATT
			CAACATCATTCCGTCCA
33	Os05g49760	192bp	TCGTCAGAAAGGTGGTG
			CCGTGAACAAGCAGAGC
